# Infection and transmission risks of COVID-19 in schools and their contribution to population infections in Germany: A retrospective observational study using nationwide and regional health and education agency notification data

**DOI:** 10.1371/journal.pmed.1003913

**Published:** 2022-12-20

**Authors:** Torben Heinsohn, Berit Lange, Patrizio Vanella, Isti Rodiah, Stephan Glöckner, Alexander Joachim, Dennis Becker, Tobias Brändle, Stefan Dhein, Stefan Ehehalt, Mira Fries, Annette Galante-Gottschalk, Stefanie Jehnichen, Sarah Kolkmann, Annelene Kossow, Martin Hellmich, Jörg Dötsch, Gérard Krause

**Affiliations:** 1 Department of Epidemiology, Helmholtz Centre for Infection Research (HZI), Braunschweig, Germany; 2 German Centre for Infection Research (DZIF), Braunschweig, Germany; 3 Chair of Empirical Methods in Social Science and Demography, University of Rostock, Rostock, Germany; 4 Hannover Medical School (MHH), Hannover, Germany; 5 Department of Pediatrics, Faculty of Medicine and University Hospital Cologne, University of Cologne, Cologne, Germany; 6 Public Health Department Konstanz, Gottmadingen, Germany; 7 Institute of Educational Monitoring and Quality Development, Agency for Schools and Vocational Training, Hamburg, Germany; 8 Public Health Department Altenburger Land, Altenburg, Germany; 9 Public Health Department Stuttgart, Stuttgart, Germany; 10 Public Health Department Cologne, Cologne, Germany; 11 Institute for Occupational, Social and Environmental Medicine, University Hospital, RWTH Aachen University, Aachen, Germany; 12 Institute of Hygiene, University Hospital of Muenster, Muenster, Germany; 13 Institute of Medical Statistics and Computational Biology, University of Cologne, Cologne, Germany; 14 TWINCORE, Centre for Experimental and Clinical Infection Research, Hannover, Germany; Edinburgh University, UNITED KINGDOM

## Abstract

**Background:**

School-level infection control measures in Germany during the early Coronavirus Disease 2019 (COVID-19) pandemic differed across the 16 federal states and lacked a dependable evidence base, with available evidence limited to regional data restricted to short phases of the pandemic. This study aimed to assess the (a) infection risks in students and staff; (b) transmission risks and routes in schools; (c) effects of school-level infection control measures on school and population infection dynamics; and (d) contribution of contacts in schools to population cases.

**Methods and findings:**

For this retrospective observational study, we used German federal state (NUTS-2) and county (NUTS-3) data from public health and education agencies from March 2020 to April 2022. We assessed (a) infection risk as cumulative risk and crude risk ratios and (b) secondary attack rates (SARs) with 95% confidence interval (CI). We used (c) multiple regression analysis for the effects of infection control measures such as reduced attendance, mask mandates, and vaccination coverage as absolute reduction in case incidence per 100,000 inhabitants per 14 days and in percentage relative to the population, and (d) infection dynamic modelling to determine the percentage contribution of school contacts to population cases. We included (a) nationwide NUTS-2 data from calendar weeks (W) 46-50/2020 and W08/2021-W15/2022 with 3,521,964 cases in students and 329,283 in teachers; (b) NUTS-3 data from W09-25/2021 with 85,788 student and 9,427 teacher cases; and (c) detailed data from 5 NUTS-3 regions from W09/2020 to W27/2021 with 12,814 cases (39% male, 37% female; median age 14, range 5 to 63), 43,238 contacts and 4,165 secondary cases for students (for teachers, 14,801 [22% male, 50% female; median age 39, range 16 to 75], 5,893 and 472). Infection risk (a) for students and teachers was higher than the population risk in all phases of normal presence class and highest in the early 2022 omicron wave with 30.6% (95% CI 30.5% to 32.6%) of students and 32.7% (95% CI 32.6% to 32.8%) of teachers infected in Germany. SARs (b) for students and staff were below 5% in schools throughout the study period, while SARs in households more than doubled from 13.8% (95% CI 10.6% to 17.6%) W21-39/2020 to 28.7% (95% CI 27% to 30.4%) in W08-23/2021 for students and 10.9% (95% CI 7% to 16.5%) to 32.7% (95% CI 28.2% to 37.6%) for staff. Most contacts were reported for schools, yet most secondary cases originated in households. In schools, staff predominantly infected staff. Mandatory surgical mask wearing during class in all schools was associated with a reduction in the case incidence of students and teachers (c), by 56/100,000 persons per 14 days (students: 95% CI 47.7 to 63.4; teachers: 95% CI 39.6 to 71.6; *p* < 0.001) and by 29.8% (95% CI 25% to 35%, *p* < 0.001) and 24.3% (95% CI 13% to 36%, *p* < 0.001) relative to the population, respectively, as were reduced attendance and higher vaccination coverage. The contribution of contacts in schools to population cases (d) was 2% to 20%, lowest during school closures/vacation and peaked during normal presence class intervals, with the overall peak early during the omicron wave. Limitations include underdetection, misclassification of contacts, interviewer/interviewee dependence of contact-tracing, and lack of individual-level confounding factors in aggregate data regression analysis.

**Conclusion:**

In this study, we observed that open schools under hygiene measures and testing strategies contributed up to 20% of population infections during the omicron wave early 2022, and as little as 2% during vacations/school closures; about a third of students and teachers were infected during the omicron wave in early 2022 in Germany. Mandatory mask wearing during class in all school types and reduced attendance models were associated with a reduced infection risk in schools.

## Abstract 2

Torben Heinsohn and co-authors investigate infection and transmission risks of COVID-19 in schools, school-level infection control measures, and their contribution to population infections in Germany.

## Author summary

**Why was this study done?:**

- Infection control policy for schools in Germany is sovereignty of the 16 federal states.
- A lack of evidence for measures in schools resulted in varying approaches to school infection control, in particular, masking and reduced attendance measures during the first year of the pandemic and beyond.
- Data for Severe Acute Respiratory Syndrome Coronavirus 2 (SARS-CoV-2) in schools was limited to small regions and short time periods.

**What did the researchers do and find?:**

- We gathered data collected by national and regional government agencies on Severe Acute Respiratory Syndrome Coronavirus 2 (SARS-CoV-2) school infections and transmission, covering March 2020 to April 2022 and in total 3,534,778 cases in students and 340,429 in staff or teachers.
- The risk of getting infected was higher for students and teachers than the general population when schools were open with all students present. Strict masking and reducing student numbers were associated with a reduction in this risk.
- Students and teachers are more likely to pass on their infection in the household than the school. Their contacts in school were responsible for up to 20% of infections in the population during the early 2022 omicron wave and as little as 2% during school closures and vacations.

**What do these findings mean?:**

- When hygiene measures to reduce infections in schools are necessary including masking policies for students and teachers in all schools during class and reducing student numbers should be considered.
- Contribution of school contacts to overall transmission in the population is highly variable, ranging from 20% during the omicron wave with open schools to 2% during closures/vacations. Limitations include underreporting of cases, misclassification of school contacts, inaccuracies in contact tracing, and lack of data on individual person factors in grouped data analysis.

## Introduction

Schools have been a key domain for nonpharmaceutical interventions (NPIs) in the Severe Acute Respiratory Syndrome Coronavirus 2 (SARS-CoV-2) pandemic. Measures include isolation and quarantine, masking and testing, hygiene measures, ventilation, and social distancing, including reduced attendance class models and school closures [1–3]. However, infection dynamics in schools, their impact on the wider population, and the effect of NPIs remain ill-defined.

In Germany, the 16 federal states hold sovereignty over education policy and in large parts health system management. During the SARS-CoV-2 pandemic in Germany, schools were first closed on March 17, 2020, nationally and gradually reopened in May 2020. Subsequently, federal states instituted diverse NPIs around social distancing, cloth masking, and reduced class sizes. Full school closures were implemented again nationally on December 16, 2020, until reopening under federal state-specific NPIs in February/March 2021. In February 2021, the German Association of Scientific Medical Societies published an evidence-based guideline on NPIs to reduce transmission of SARS-CoV-2 in schools [4]. The variability of measures between federal states diminished but remained in some parts such as masking of primary school students or masking of students during class. Different testing strategies (e.g., antigen testing, pooled PCR testing, mandatory/voluntary) for students and teachers were implemented in spring 2021. For a third time school closures, now depending on local infection dynamics, were instituted in April 2021 along with mandatory testing in schools nationally. All federal states returned to face-to-face classes and reduced stringency of measures prior to the summer vacation 2021. In September 2021, the adult population in Germany surpassed a vaccination coverage of 60% [5]. The National Advisory Committee for Immunisations (Ständige Impfkommission, STIKO) issued a vaccine recommendation for adolescents from 12 to 17 years of age and for children at risk in June 2021 and a general recommendation for all adolescents in August 2021 [6]. Despite increasing case numbers, schools remained in normal attendance models throughout the winter of 2021/2022. Comparing this internationally, after the first lockdown of 2020 approaches have differed. Whereas some centralised countries such as France have opted to avoid school closures thereafter, other such as the United Kingdom have continued to use it as a measure. Other nations with a federal structure such as the United States have seen a similar plethora of highly localised approaches. This illustrates both varying political approaches to pandemic management, while highlighting the lack of an evidence base that can unite different political factions behind one common, evidence-based approach.

Whereas school closures have proven effective in containing SARS-CoV-2 transmission [7–9], they are associated with adverse effects on the physical and mental health of students and exacerbate ethnic and socioeconomic disparities [10–14]. Legislators implemented NPIs and testing in schools to reduce setting specific transmission [15] and avoid closures. Evidence of effects of measures was initially derived from modelling studies or indirectly from non-school settings [3]. The sovereignty of federal states over education policy resulted in 16 different and parallel approaches. This allows for comparative evaluation of infection dynamics and the effects of NPIs in schools.

The risk of infection and transmission in schools relative to the population remain contentious. In a systematic review [16], we showed that during phases with low population incidence, there is no increased risk of infection in students or school staff; however, with increasing infection dynamics in the population, the risk of infection in students and staff increases. In Germany and Europe, studies on school transmission risk and contribution to overall transmission in the population are based on geographically and temporally limited data [3,17–20], with few exceptions [21]. Results do not show longitudinal changes in the same dataset, neither do reports from seroprevalence studies on infection risk in students [15]. As these parameters change with pandemic development and advent of new variants, our study covers a period of over 2 years to deliver interval-specific outcomes. Furthermore, no studies describing infection risk and contribution of schools for the omicron variant were identified. Analysing the role of schools in the pandemic, current studies mostly remain descriptive and do not account for age- and setting-specific infection dynamics and underdetection of cases [18–20,22]. Both setting-specific transmission risk as well as setting specific contribution to overall infections is crucial to inform pandemic policy on effects of NPIs for schools. Lastly, estimates of effect sizes of measures are predominantly derived from modelling studies [15,23] or comparing large diverse regions or different times during the pandemic [15,23].

In this study, we obtained federal state and county-level data for school settings as well as detailed regional data obtained from individual public health and educational agencies. We investigated (a) the risk of infection in students and staff over time and relative to the population; (b) the risk of transmission of students and staff in schools and other contact settings; (c) the effect of NPIs in schools on infection incidence in students and staff; and (d) the contribution of contacts in schools to the overall population infections during the first 24 months of the pandemic in Germany.

## Methodology

### Study design and data sources

First, we performed a retrospective observational study of prospectively collected SARS-CoV-2 infection case report data from health and educational authorities in Germany (Ethical approval N°9609_BO_K_2021, Hannover Medical School) from 5 regions. Public health authorities report case data according to the Infection Protection Act (IfSG). Cases were defined by direct viral detection via nasopharyngeal swabs using PCR or by cultural isolation of the pathogen. In addition to notified cases, some regional data included secondary cases as well as contacts per case, allowing to compute secondary attack rates (Text A and B in S1 File). Data were provided for individual student or staff units.

Secondly, we used data collected by the Standing Conference of Ministers of Education and Cultural Affairs in Germany (KMK) [24]. Federal state (NUTS-2) agencies collected data weekly in a structured format from all schools within their jurisdiction and included the aggregated number of students, classes, and staff infected or absent due to quarantine and school closures. Federal state-level (NUTS-2) data are publicly available from calendar week (W) 46/2020 to W15/2022 onwards, excluding school closures and vacations. The analysis of this data was expanded into the omicron era in response to reviewers’ comments. In addition, we received county-level (NUTS-3) data, collected in the same manner on the same parameters (Table 1 of Text A in S1 File) [25].

Thirdly, we sourced population infection and incidence data from the Robert Koch Institute (RKI) SURVSTAT tool, described as incidence per 100,000 inhabitants or raw case numbers [5](Fig A in S1 File). Data are aggregated as infections per geographical and time unit. We also sourced time and county-specific data on hygiene and testing measures in schools from government sources (Tables A–E in S1 File).

As calendar weeks (W) according to ISO8601 are the main time unit in German state agencies, we conducted the analysis also in calendar weeks. This study is reported as per the Strengthening the Reporting of Observational Studies in Epidemiology (STROBE) guideline (S1 STROBE Checklist). The study protocol can be found in S1 Study Protocol, which outlines analysis of infection and transmission risks as well as the effects of measures. The plan for analysis of the contribution to population infections was added after protocol approval.

### Data analysis

We performed (a) a descriptive analysis of infection risk for students and staff and/or teachers as cumulative risks as percentages (aggregated cases in a population over a defined period of time) on data grouped by students or staff by age, school form, and time period with 95% confidence intervals (CIs) and crude risk ratios of student and teacher cumulative risk relative to the population of the same geographical unit (NUTS-2). We (b) calculated secondary attack rates (SARs) as percentages with 95% CI of those being identified as notified infections in all known contacts on data grouped by students or staff by age, school form, and time period. We calculated CIs for binomial values using the Agresti–Coull approach [26]. Data were analysed in R Studio (v1.3.959 with R v4.0.1) for aims (a) and (b), in STATA (v14) for aim (c). The model for aim (d) was developed in Python (v3.8.1).

We adapted the pandemic phases proposed by the RKI [5] for school policies (Table 1).

**Table 1:** Overview of the pandemic phases and outline of school policies.

Phase	Duration	Infection environment	School policy environment
1	W10-W20/2020	First COVID-19 wave in Germany	- W12-17 school closures - W18+ phased reopening with hygiene concepts
2	W21-39/2020	Summer Plateau 2020	- W21+ phased reopening with hygiene concepts - Summer vacation - Normal face-to-face class with sporadic cloth mask rules introduced
3	W40/2020–08/2021	Second COVID-19 wave	- W40-51 face-to-face classes with tightening cloth mask rules - W52-02 Christmas vacation - W02-08 school closures
4	W09-23/2021	Third COVID-19 wave Variant of concern: alpha	- W09+ reduced attendance class models with surgical mask rules, gradual introduction of testing, teachers given priority for vaccinations - W16+ *Bundesnotbremse* with incidence-dependent school closures, mandatory testing - W21+ phased return to face-to-face classes
5	W24-30/2021	Summer Plateau 2021	- Face-to-face class with loosening of mask and testing mandates, summer vacation - W30 vaccination coverage in the total population reaches 50%
6	W31-51/2021	Fourth COVID-19 wave Variant of concern: delta	- W33 vaccinations started for adolescents age 12 to 17 years - Retightening of mask and testing mandates - W49 vaccinations for 5–11-year-olds with comorbidities recommended
7	W52/2021	Fifth COVID-19 Variant of concern: omicron	- Daily testing in schools in some states, continued presence classes
**Adaptations made for this study**			
3a	W40-51/2020	Second COVID-19 wave	- W40-51 face-to-face classes with tightening cloth mask rules
3b	W52/2020 –W07/2021	Second COVID-19 wave	- W52-02 Christmas vacation - W02-08 school closures
3 to 4	W07-08/2021		- School reopening from W08/2021

Based on Schilling and colleagues [27], own illustration and additions. W = calendar week. *Bundesnotbremse* was a law passed in March 2021 to enable nationwide standardisation of infection control measures above a specific 7-day case incidence unifying 16 separate federal state approaches for its duration, including reduced attendance and school closures as well as mandatory biweekly testing in schools.

We (c) investigated infection control measures and other factors influencing infection risk of aggregated teachers or students in a multiple linear regression model, using incidence as in notified SARS-CoV-2 infections per 100,000 teachers and students over 14 days per county as the outcome variable. Incidence over 14 days was chosen as the NUTS-3 data from KMK registries used includes active cases mostly, of which we assume that most will be going back to school after 2 weeks according to isolation guidance to reduce counting cases double. We included an explanatory variable on whether schools were closed due to vacation, mostly closed due to official school closures (but with emergency care in place), partly closed (with some classes being on site), or not closed. We used a random effects multilevel GLS model to test for statistical associations of the official active cases per 100,000 students and teachers as reported by the KMK for the county level on the 2-week lags of the attendance rates, the stringency of mask mandates (Table 1 of Text B in S1 File) and the mandate and stringency of testing in schools. The data were set to panel data by using calendar weeks as a time and county as a regional variable. We controlled for the 2-week incidence per 100,000 inhabitants of the corresponding county, the percentage of fully vaccinated persons in the corresponding federal state, the socioeconomic status, and geography of the county (Text B in S1 File). Furthermore, we tested whether the school-specific NPIs had an over proportional effect on the school population compared to the overall population. We proceeded similarly to the first approach, yet did not include a control group as an explanatory variable but instead divided the active cases among the students or teachers, respectively, by the 14-day incidence of the total population of the district. This way, we estimated the effect of the NPIs on cases in schools relative to cases in the population, thus estimating whether the NPIs in schools have a significantly different effect on the overall population as on the school population.

**Fig 1:**
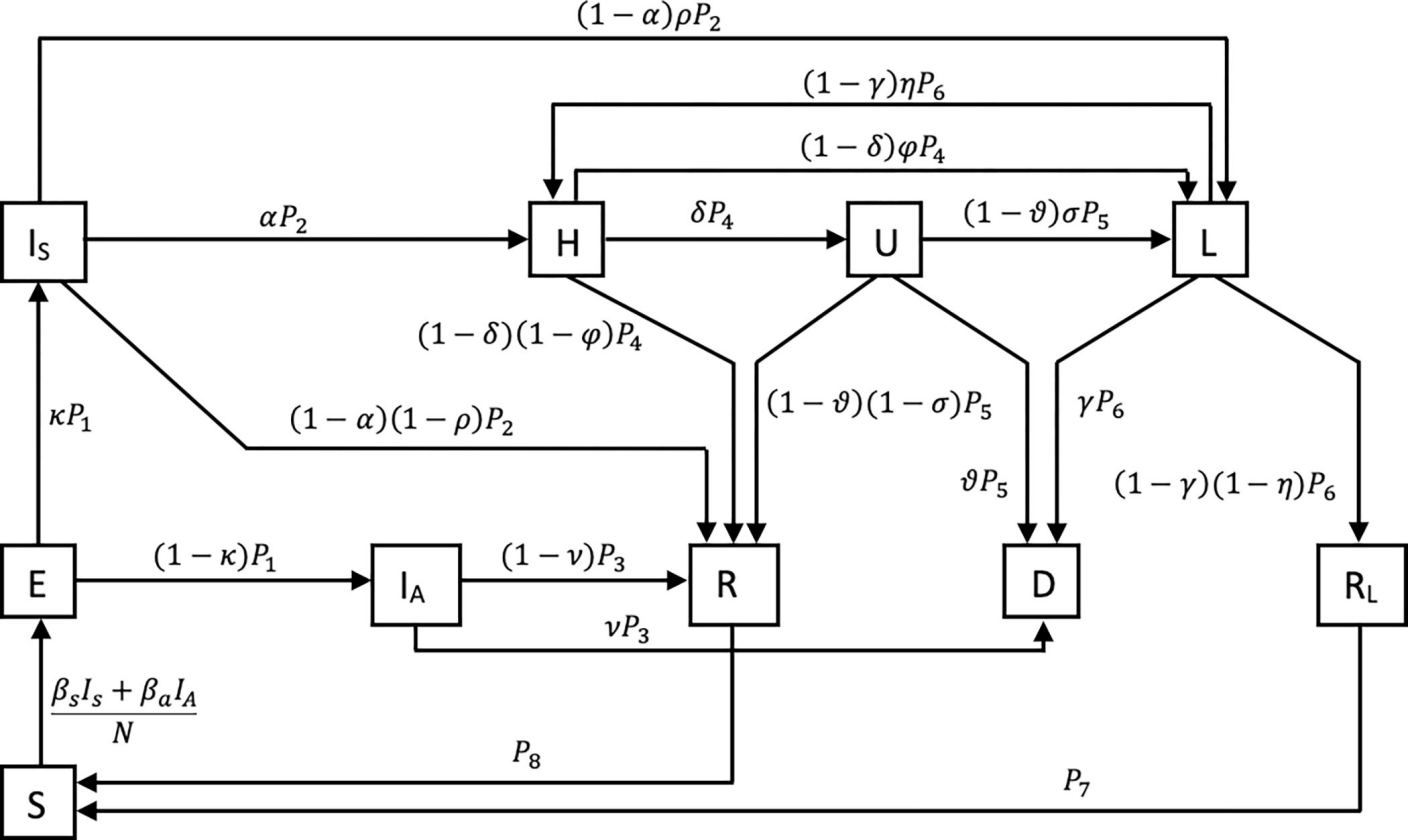
Compartmental SEIR model. Legend: An individual in age group i is classified either as susceptible (S), exposed (E), asymptomatically infected (I_A_), symptomatically infected (I_S_), hospitalised (H), in intensive care (U), suffering under long-COVID (L), fully recovered (R_F_), recovered from long-COVID (R_L_), and dead (D). P_1_−P_8_ are health state transition rates. The Greek letters quantify transition probabilities between the statuses.

We (d) estimated infection dynamics in schools and their contribution to population infections using a SEIRS (Susceptible-Exposed-Infectious-Recovered-Susceptible) model previously described [28]. This distinguishes between healthy but susceptible individuals, those infected but not yet infectious (exposed), and symptomatic and asymptomatic patients. In addition, we included compartments for hospitalisations, patients entering intensive care units (ICUs) and persons with long-COVID, i.e., those who continue to have sequelae after recovery. In the final state, the patients are recovered or dead. Furthermore, we assume a reinfection process. We split the recovery compartment into a compartment for those recovered from COVID and a long-COVID compartment, since we assume that both have a different reinfection rate. For the school context, we use aggregated student and teacher cases and divided the population into 9 age groups with known contact matrices and differentiated between school and non-school contacts. The variables describing time-dependent risk of infection per contact in a given age group were adapted to scaling parameters, scaling the respective contact matrix to reflect changes due to interventions or behavioral change (Text B in S1 File). Fig 1 illustrates the model as a flow chart.

The remaining parameters represent time periods of transitions between the different statuses as well as probabilities for the transitions and are estimated from international data and literature research. The data for the model is composed of reports from KMK [24], RKI [5], and the DIVI Intensive Care Register [29]. We accounted for age-specific underdetection taking pandemic period- and age-specific underdetection ratios from a large seroprevalence study in Germany [30] for variants prior to omicron (research ongoing).

## Results

### Data characteristics

We obtained data from 5 regional agencies covering 3 urban and 2 rural NUTS-3 regions. We used data from the KMK on the federal state (NUTS-2) level covering 16/16 federal states and data on the county (NUTS-3) level with data on 275 out of 401 counties. For clarity, Table 2 provides an overview of data sources used with case and contact numbers and demographics and in which analyses data was used.

**Table 2:** Data sourced and outcomes.

	Data provided by 5 local authorities	Data provided by the Standing Conference of Ministers of Education and Cultural Affairs (KMK)	
Location	5 NUTS-3 regions	275/401 NUTS-3	16/16 NUTS-2
Time from	W10/2020	W09/2021	(1) W46/2020 (2) W08/2021
Time until	W27/2021	W25/2021	(1) W50/2020 (2) W15/2022
Students	545,409	5,092,252	10,103,054
Staff (teachers)	60,401	(624,944)	(911,360)
Cases	15,433	95,215	3,851,247
Male (%); female (%) age median (range) interquartile range	35%; 35%* 15 (5–75) 11–18	NA	NA
Contacts	49,131	NA	NA
Male (%); female (%) age median (range) interquartile range	49%; 50% 16 (0–103) 10–32	NA	NA
Used for outcomes	(a) Risk of infection (cumulative risk, subgroup analysis) (b) Risk of transmission (secondary attack rates)	(a) Risk of infection (cumulative risk, crude risk ratios) (c) Effect of infection control measures	(a) Risk of infection (cumulative risk) (d) Contribution of contacts in schools to population infections

Overview of data sources and mapping to outcomes.

*Remaining percentage of cases did not have gender specified in the source data. For more detailed overview of data sources see Text A in *S1 File*.

### Risk of infection for students and teachers in Germany (a)

Data obtained from KMK on the federal state (NUTS-2) level from W46-50/2020 and W08/2021 to W15/2022 covered 3,521,964 student and 329,913 teacher cases. Cumulative risk of notified SARS-CoV-2 infection for students was 0.96% (95% CI 0.95% to 0.97%) in W46-50/2020, 1.22% (95% CI 1.21% to 1.23%) in phase 4 (variant of concern (VOC): alpha), 0.14% (95% CI 0.13% to 0.14%) in phase 5 (summer plateau), 9.75% (95% CI 9.73% to 9.78%) in phase 6 (VOC: delta), and 30.58% (95% CI 30.54% to 30.61%) in phase 7 (VOC: omicron). For teachers, risk of infection was 1.77% (95% CI 1.74% to 1.79%) in W46-50/2020, 1.53% (95% CI 1.50% to 1.55%) in phase 4, 0.1% (95% CI 0.09% to 0.11%) in phase 5, 6.11% (95% CI 6.05% to 6.16%) in phase 6, and 32.66% (95% CI 32.55% to 32.76%) in phase 7, respectively.

County (NUTS-3) level data by the KMK for W09-25/2021 showed an infection risk of 1.68% (95% CI 1.67% to 1.7%) for students and 1.51% (95% CI 1.48% to 1.54%) for teachers (Table 3).

**Table 3:** Risk of infection over pandemic phases with minima and maxima risk per federal state/county according to KMK data.

		Standing Conference of the Ministers of Education and Cultural Affairs (KMK) Data																	
		NUTS-2 (federal state level)															NUTS-3 (county level)		
		(W46-50/2020 only)			Phase 4 (W08-23/21)			Phase 5 (W24-30/21)			Phase 6 (W31-51/21)			Phase 7 (W52-15*)			(W09-25/2021)		
		Reg ion	Cases/Pop.	Risk (CI)	Reg ion	Cases/Pop.	Risk (CI)	Reg ion	Cases/Pop.	Risk (CI)	Reg ion	Cases/Pop.	Risk (CI)	Reg ion	Cases/Pop.	Risk (CI)	Region	Cases/Pop	Risk (CI)
**Students**																			
Infection Risk	Min	MV	126/163,600	0.08% (0.06% to 0.09%)	SH	754/435,539	0.17% (0.16% to 0.19%)	SH	3/435,539	0.001% (0% to 0.002%)	HE	11403/760,400	1.50% (1.47% to 1.53%)	HB	6810/72,092	9.45% (9.23% to 9.66%)	01057	15/13,925	1.1% (1% to 1.2%)
	Max	BE	6,595/400,113	1.65% (1.61% to 1.69%)	RP	11,804/520,712	2.27% (2.23% to 2.31%)	BY	3,431/1,497,783	0.23% (0.22% to 0.24%)	SN	74,490/412,957	18.15% (18.03% to 18.30%)	ST	69,664/185,838	37.49% (37.27% to 37.70%)	07319	443/12,131	3.6% (3.5% to 3.7%)
**Teachers**																			
Infection risk	Min	SH	96/48,500	0.2% (0.16%-0.24%)	SH	57/48,500	0.12% (0.09% to 0.15%)	SH	0/ 48500	0.07% (0.05% to 0.10%)	HE	672/61,931	1.09% (1.01% to 1.17%)	HB	473/ 9,151	5.17% (4.73% to 5.64%)	03154	0/829	0% (0% to 0.8%)
	Max	BE	2101/44,000	4.78% (4.58% to 4.98%)	TH	3,190/21,072	4.21% (3.95% to 4.49%)	BY	395/162,218	0.24% (0.22% to 0.27%)	ST	2,548/14,188	17.96% (17.34% to 18.60%)	ST	6395/13,541	47.23% (46.39% to 48.10%)	16,063	75/875	8.6% (8.5% to 8.6%)

BE = Berlin, HB = Bremen, HE = Hessen, MV = Mecklenburg Western Pommerania, RP = Rhineland Palatinate, SH = Schleswig Holstein, ST = Saxony Anhalt, SN = Saxony, 01057 = Plön, 07319 = Worms, 03154 = Helmstedt, 16063 = Wartburgkreis. Cases/Pop. = Nominator of reported cases/denominator of relevant population.

*W15 last week of data reporting. Berlin stopped reporting in W5/2022; 95% confidence intervals in brackets. Minima (min) and maxima (max) represent the highest and lowest risk of infection observed across all regions during the given time interval. Full data in Table G in S1 File.

Quarantine risk of students and teachers over federal states varied widely from 0.19% (95% CI 0.15% to 0.23%) to 37.13% (36.48% to 37.79%) over time.

Data obtained from 5 regional agencies covered 15,433 index cases, 49,131 contacts, and 4,637 secondary cases reported during the school year 2020/2021 across 5 regions of Germany. Among index cases, 35% were male, 35% female, and median age was 15 (5 to 75).

Infection risk in phases 2 to 4 ranged from 2.04% (95% CI 2.02% to 2.06%) to 7.6% (95% CI 7.4% to 7.8%) for the general population, 1.2% (95% CI 1.16% to 1.25%) to 5.6% (95% CI 5.1% to 6%) for students, and 2.1% (95% CI 1.9% to 2.2%) to 3.2% (95% CI 3.1% to 3.4%) for staff (Table 4).

**Table 4:** Risk of infection over pandemic phases with minima and maxima risk per federal state/county according to regional agency data.

		Regional agency data											
		Phase 2 (W21-39/2020)			Phase 3a (W40-51/2020)			Phase 3b (W52/2020-7/2021)			Phase 4 (W8-23/2021)		
		Reg.	Cases/Pop.	Infection risk	Reg.	Cases/Pop.	Infection risk	Reg.	Cases/Pop.	Infection risk	Reg.	Cases/Pop.	Infection risk
**All students**	Min	R4	27/34,369	0.08% (0.05% to 0.11%)	R4	127/34,369	0.37% (0.31% to 0.44%)	R4	121/34,369	0.35% (0.29% to 0.42%)	R4	225/34,369	0.65% (0.57% to 0.75%)
	Max	R5	195/94,350	0.21% (0.18% to 0.24%)	R5	1,529/94,350	1.62% (1.54% to 1.70%)	R3	162/10,046	1.61% (1.38% to 1.88%)	R3	297/10,046	2.96% (2.64% to 3.31%)
Age <10	Min	R3	1/3,485	0.03% (0% to 0.18%)	R4	24/8,884	0.27% (0.18% to 0.40%)	R1	120/38,207	0.31% (0.26% to 0.38%)	R4	77/8,884	0.87% (0.69% to 1.08%)
	Max	R5	46/26,252	0.21% (0.18% to 0.24%)	R5	253/26,252	0.96% (0.85% to 1.10%)	R3	34/3485	0.98% (0.69% to 1.36%)	R3	100/3,485	2.87% (2.36% to 3.48%)
Age 10–14	Min	R3	3/3,510	0.09% (0.02% to 0.26%)	R4	74/12,681	0.58% (0.46% to 0.73%)	R4	37/12,681	0.29% (0.21% to 0.4%)	R4	96/12,681	0.76% (0.62% to 0.92%)
	Max	R5	74/33,941	0.22% (0.17% to 0.27%)	R5	518/33,941	1.53% (1.40% to 1.70%)	R3	42/3,510	1.20% (0.88% to 1.62%)	R3	93/3,510	2.65% (2.17% to 3.24%)
Age 15–19	Min	R4	4/12,804	0.03% (0.01% to 0.08%)	R4	35/12,804	0.27% (0.20% to 0.38%)	R1	236/42,356	0.56% (0.49% to 0.63%)	R4	52/12,804	0.41% (0.31% to 0.53%)
	Max	R1	104/42,356	0.25% (0.2% to 0.3%)	R5	758/34,157	2.22% (2.07% to 2.38%)	R3	86/3,051	2.82% (2.29% to 3.47%)	R3	102/3,051	3.34% (2.76% to 4.04%)
Primary school	Min	R2	41/76,768	0.05% (0.04% to 0.07%)	R2	484/76,768	0.63% (0.58% to 0.69%)						
	Max	R1	54/37,799	0.14% (0.11% to 0.19%)	R1	346/37,799	0.92% (0.82% to 1.02%)	R1	132/37,799	0.35% (0.29% to 0.41%)	R1	609/37,799	1.61% (1.49% to 1.74%)
Secondary school	Min	R2	180/120,206	0.15% (0.13% to 0.17%)	R2	1,446/120,206	1.20% (1.14% to 1.27%)						
	Max	R1	118/62,821	0.19% (0.16% to 0.23%)	R1	1,059/62,821	1.69% (1.59% to 1.79%)	R1	360/62,821	0.57% (0.52% to 0.64%)	R1	1,167/62,821	1.86% (1.75% to 1.97%)
Vocational school	Min	R2	68/55,021	0.12% (0.1% to 0.16%)	R2	638/55,021	1.16% (1.07% to 1.26%)						
	Max	R1	77/40,926	0.19% (0.15% to 0.24%)	R1	494/40,926	1.21% (1.11% to 1.32%)	R1	108/40,926	0.26% (0.22% to 0.32%)	R1	341/40,926	0.83% (0.75% to 0.93%)
Spec. needs school	Min	R2	4/4,570	0.09% (0.03% to 0.23%)	R1	58/4,516	1.28% (0.99% to 1.66%)						
	Max	R1	9/4,516	0.20% (0.10% to 0.38%)	R2	62/4,570	1.36% (1.06% to 1.74%)	R1	23/4,516	0.51% (0.34% to 0.77%)	R1	82/4,516	1.82% (1.46% to 2.25%)
**All Staff**	Min	R2*	22/22,807	0.1% (0.06% to 0.15%)	R1	670/38314	1.75% (1.62–1.88%)						
	Max	R1	87/38,314	0.23% (0.18% to 0.28%)	R2*	425/22,807	1.86% (1.70% to 2.05%)	R1	161/38,314	0.42% (0.36% to 0.49%)	R1	311/38,314	0.81% (0.73% to 0.91%)
Age <30		R1	11/3,755	0.18% (0.01% to 0.33%)	R1	153/3,755	2.51% (2.14% to 2.93%)	R1	25/3,755	0.41% (0.27% to 0.61%)	R1	61/3,755	1% (0.77% to 1.28%)
Age 30–34		R1	22/6,053	0.36% (0.24% to 0.55%)	R1	107/6,053	1.77% (1.46% to 2.13%)	R1	30/6,053	0.50% (0.34% to 0.71%)	R1	45/6,053	0.74% (0.55% to 1%)
Age 35–39		R1	15/5,632	0.27% (0.16% to 0.44%)	R1	83/5,632	1.47% (1.19% to 1.82%)	R1	22/5,632	0.39% (0.25% to 0.59%)	R1	45/5,632	0.80% (0.60% to 1.07%)
Age 40–44		R1	12/5,172	0.23% (0.13% to 0.41%)	R1	87/5,172	1.68% (1.36% to 2.07%)	R1	22/5,172	0.43% (0.28% to 0.65%)	R1	46/5,172	0.89% (0.66% to 1.19%)
Age 45–49		R1	14/5,402	0.26% (0.15% to 0.44%)	R1	72/5,402	1.33% (1.06% to 1.68%)	R1	17/5,402	0.31% (0.19% to 0.51%)	R1	47/5,402	0.87% (0.65% to 1.16%)
Age 50–54		R1	5/4,253	0.12% (0.04% to 0.28%)	R1	75/4,253	1.76% (1.41% to 2.21%)	R1	21/4,253	0.49% (0.32% to 0.76%)	R1	30/4,253	0.71% (0.49% to 1.01%)
Age 55–59		R1	3/4,406	0.07% (0.01% to 0.21%)	R1	55/4,406	1.25% (0.96% to 1.62%)	R1	15/4,406	0.34% (0.2% to 0.57%)	R1	23/4,406	0.52% (0.34% to 0.79%)
Age >60		R1	4/3,640	0.11% (0.03% to 0.29%)	R1	38/3,640	1.04% (0.76% to 1.43%)	R1	9/3,640	0.25% (0.12% to 0.48%)	R1	13/3,640	0.36% (0.20% to 0.62%)
Primary school	Min	R2*	8/6,214	0.13% (0.06% to 0.26%)	R2*	123/6,124	2.01% (1.68% to 2.39%)						
	Max	R1	31/10,715	0.29% (0.20% to 0.41%)	R1	267/10,715	2.49% (2.21% to 2.80%)	R1	43/10,715	0.40% (0.3% to 0.54%)	R1	106/10,715	0.99% (0.82% to 1.20%)
Secondary school	Min	R2*	9/11,296	0.08% (0.04% to 0.15%)	R1	250/18,927	1.32% (1.17% to 1.49%)						
	Max	R1	33/18,927	0.17% (0.12% to 0.25%)	R2*	251/11,296	2.22% (1.97% to 2.51%)	R1	76/18,927	0.40% (0.32% to 0.5%)	R1	125/18,927	0.66% (0.55% to 0.79%)
Vocational school	Min	R2*	1/2,722	0.04% (0% to 0.23%)	R2*	23/2,722	0.84% (0.57% to 1.27%)						
	Max	R1	12/4,810	0.25% (0.14% to 0.44%)	R1	67/4,810	1.39% (1.1% to 1.77%)	R1	13/4,810	0.27% (0.15% to 0.47%)	R1	31/4,810	0.64% (0.45% to 0.92%)
Spec. needs school	Min	R1	9/3,591	0.25% (0.12% to 0.48%)	R1	81/3,591	2.26% (1.82–2.80%)						
	Max	R2*	4/925	0.43% (0.13% to 1.15%)	R2*	27/925	2.92% (2% to 4.23%)	R1	22/3,591	0.61% (0.40% to 0.93%)		36/3,591	1% (0.72% to 1.39%)

*Teachers only. 95% confidence intervals in brackets. Spec. needs schools = special needs schools. R / Reg. = Region. Cases/Pop. = Nominator of reported cases/denominator total of relevant population. Minima (min) and maxima (max) represent the highest and lowest cumulative risk of infection (cases in a population over the population total) observed across all 5 regions during the given time interval. Fields with a single value indicate that data was only available from one region. For detailed regional data see Table F in S1 File.

**Fig 2:**
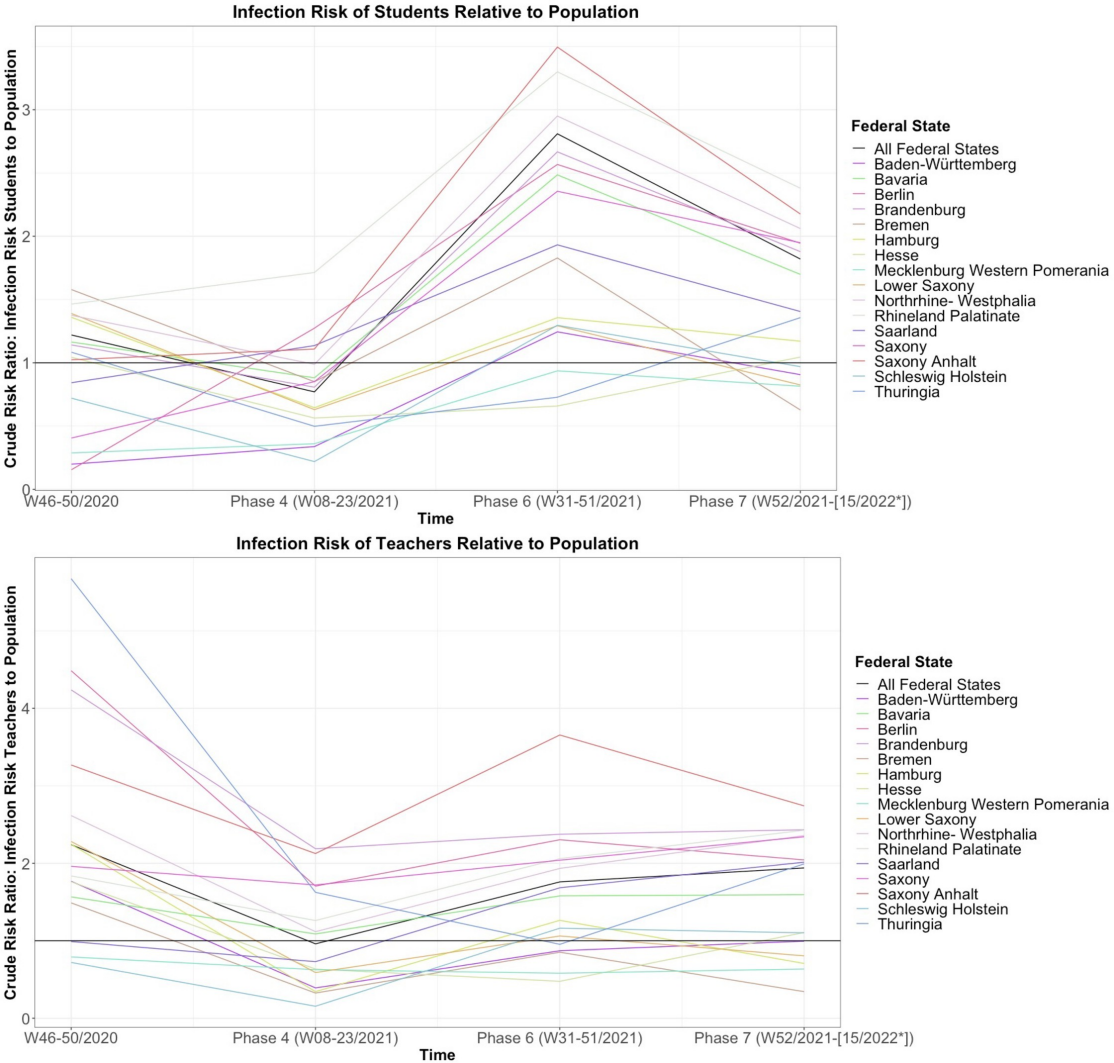
CRR of (A) students to population and (b) teachers to population. CRR of infection of students (A) and teachers (B) compared to the infection risk of the population in the respective federal state and time interval, based on NUTS-2 level data by the KMK. A crude risk ratio of 1 indicates equal risk of infection. *End of phase 7 not yet defined, data collection by KMK ceased W15/2022. As phase 5 covers the summer plateau and summer vacation it has not been included in this graphic due to data paucity. All data in Table G in S1 File. Phase 4 VOC alpha, phase 6 VOC delta, phase 7 VOC omicron. CRR, crude risk ratio; VOC, variant of concern.

Based on NUTS-2 level KMK data, on the federal state level, the crude infection risk ratio (CRR) for students to the population showed a risk similar or lower to the general population in W46-50/2020. It was lower in phase 4 and rose to its peak in phase 6, where it exceeded that of the general population, and remained higher in phase 7 (Fig 2A). For teachers, the CRR ratio was highest in W46-50/2020. It was lower in phase 4 and higher than the general population in phases 6 and 7 (Fig 2B). The CRR relative to the population was higher for teachers than students in W46-50/2020 and higher for students than teachers in phase 6.

**Fig 3:**
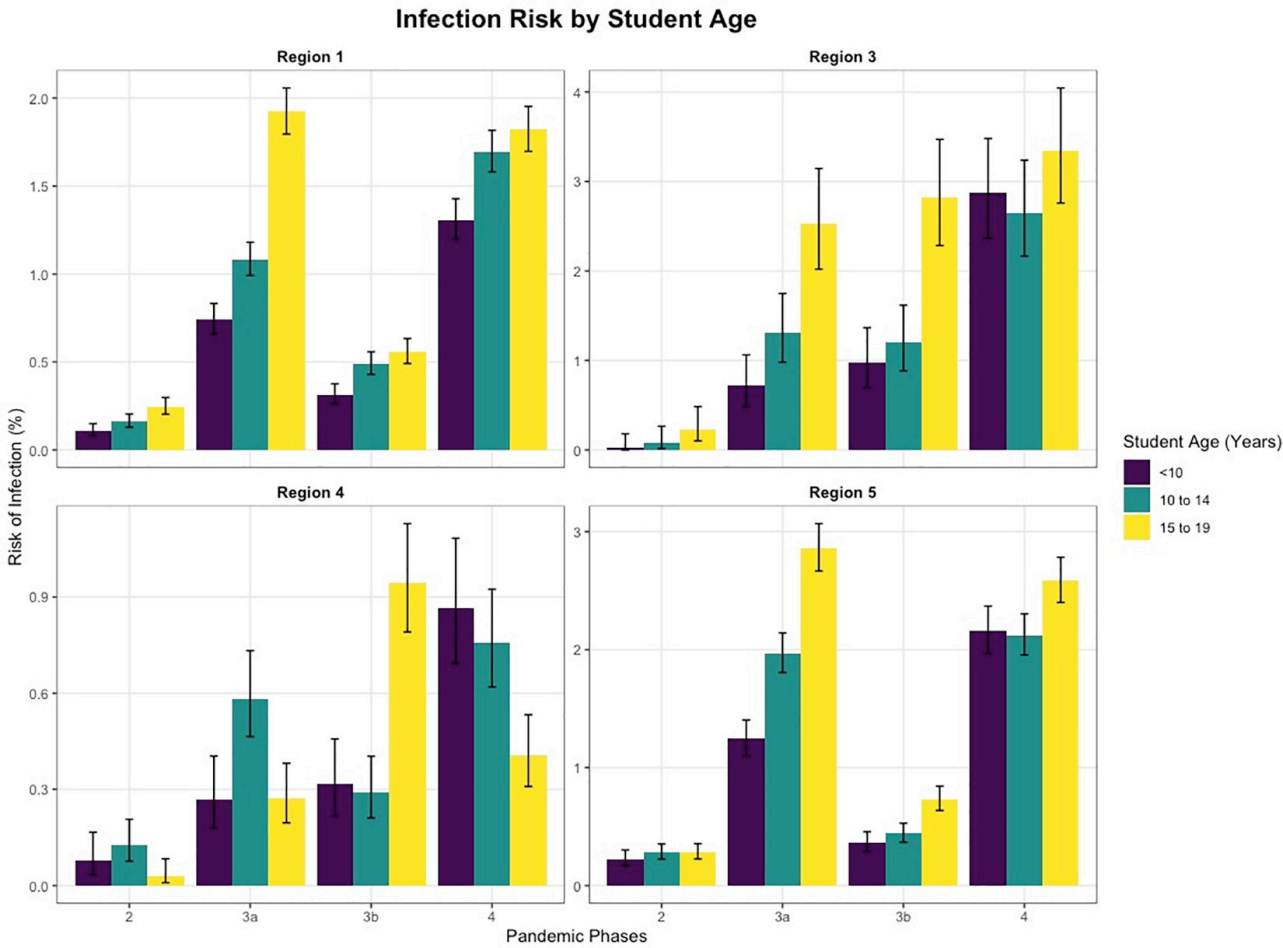
Cumulative risk of infection of students by age over time in 4 regions. Error bars show CI for risk of infection. Based on detailed data by 5 regional agencies. Phase 2 summer plateau 2020, phase 3a second wave with open schools, phase 3b second wave with school closures, phase 4 mixed class models and VOC alpha. CI, confidence interval; VOC, variant of concern.

Based on data from 5 regional agencies, infection risk was lower for students and staff than the general population during periods of school closure in particular (Figs B–I in S1 File). Student infection risk increased with student age. The under 10-year-olds showed a lower risk of infection than the 10-to-14 and 15-to-19-year-olds in most phases and regions (Fig 3). Infection risk was higher for students in school forms with an older student population. Staff infection risk was similar across age groups. There was a trend towards higher infection risk in special needs and primary school staff and lower risk in secondary school staff (Text C in S1 File).

### Risk of transmission after contact with a notified case (secondary attack rates) (b)

Based on contact tracing data provided by regional agencies, overall risk of infection after contact with a notified case (SAR) for the observation period ranged from 4.6% (95% CI 3.8% to 5.6%) to 12.8% (95% CI 12.3% to 13.3%) across 4 regions. For the different phases, ranges were 4.1% (95% CI 3.3% to 5%) to 16.7% (95% CI 1.1% to 58.2%) (phase 2), 4.9% (95% CI 4.5% to 5.3%) to 9.1% (95% CI 8.5% to 9.7%) (phase 3a), 5.3% (95% CI 2.4% to 10.8%) to 19.4% (95% CI 16.9% to 22.2%) (phase 3b), and 5.4% (95% CI 4.2% to 6.9%) to 19% (95% CI 17.9% to 20.1%) (phase 4) (Table H in S1 File).

SAR increased with index and contact age; 15-to-19-year-old student index cases had a higher SAR than under 10-year-olds and mostly higher than 10-to-14-year-olds. SARs increased with contact age and for school forms with an older student population (Text D in S1 File).

**Fig 4:**
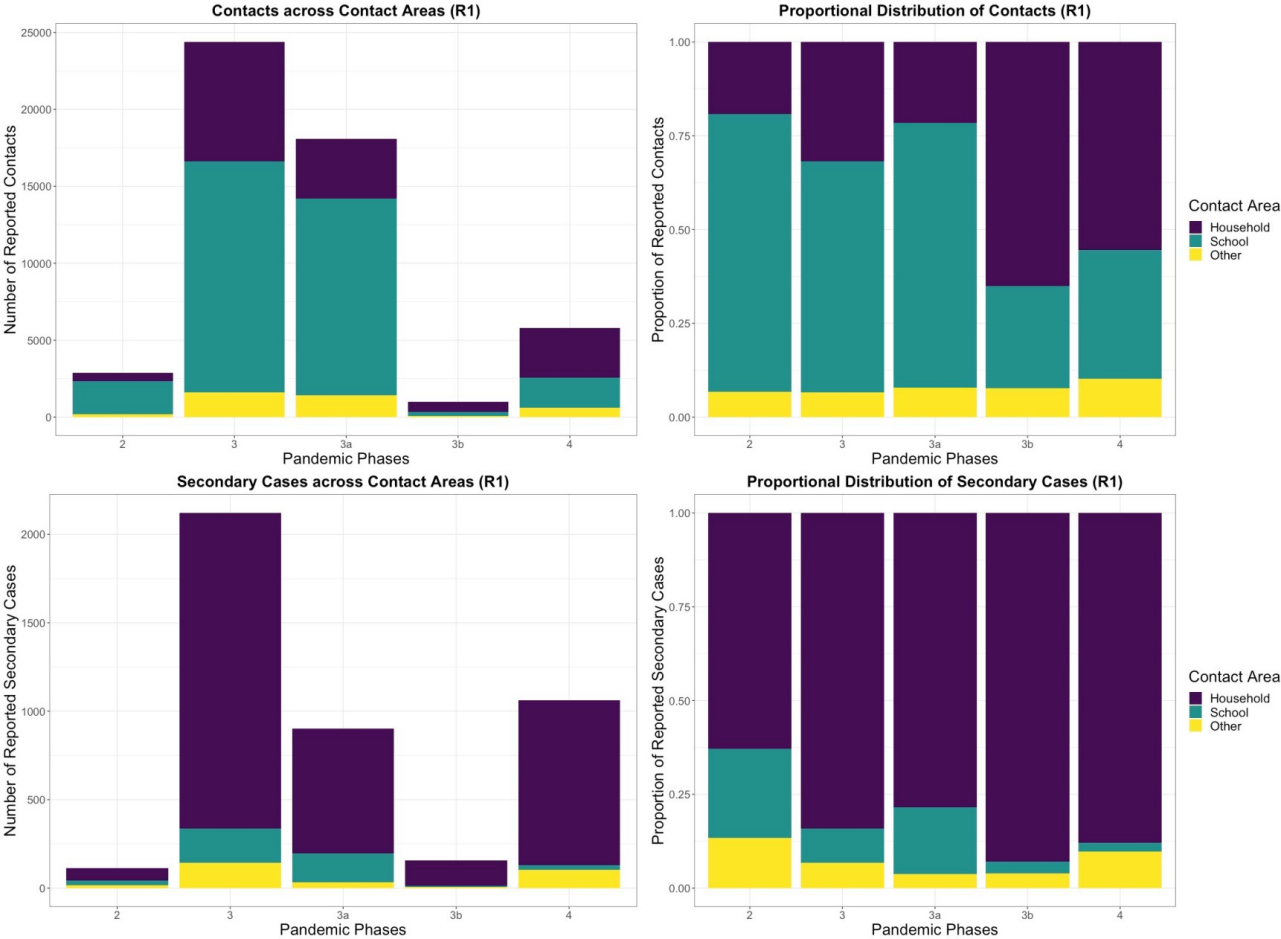
Contacts and secondary cases in phases 2–4 (W21 2020 to W23 2021) (R1). Total amounts of contacts (top left) and secondary cases (bottom left) reported by students in R1. The proportions of household, school, and other contact areas to the number of contacts (top right) and secondary cases (bottom right) and their change over time and policy environments are shown. Phase 2 represents open schools with low population incidence and low measures, phase 3a a time of high incidence and open schools with few measures, phase 3b is an interval of school closures, phase 4 shows reduced attendance models with tightened measures and VOC alpha (see Table 2). VOC, variant of concern.

Data from 1 region allows differentiation of SARs by contact settings. Overall, for students, SAR was 8.2% (95% CI 7.8% to 8.6%), with 1.2% (95% CI 1.1% to 1.4%) for contacts in schools, 23.2% (95% CI 22.2% to 24.2%) for households, and 8.4% (95% CI 7% to 10%) for other contact areas. Overall, SAR for staff was 8% (95% CI 7.4% to 8.7%), with 1.5% (95% CI 1.2% to 2%) in schools, 19.6% (95% CI 17.8% to 21.4%) in households, and 7.3% (95% CI 5.4% to 9.7%) in other contact areas. For students, most contacts (64%, for staff 57%) were recorded for schools as the contact area; most secondary cases (84%, for staff 80%) were reported in household contacts (Fig 4). In total, 2.2 times more contacts were reported in schools, whereas 8.8 times more secondary cases originated in households relative to schools.

Differences in total SAR between phases of open and closed schools arose from changes in proportions of contacts from different contact areas. For students, with similar school and household SARs, overall SAR changed from 4.9% (95% CI 4.5% to 5.3%) in phase 3a (open schools) with 74.8% and 20% of contacts reported for schools and household, respectively, to 15.5% (95% CI 13% to 18.4%) SAR in phase 3b (school closure) with household contacts at 65.6% (Fig 4). During this interval, average reported contacts were 10.6 (phase 3a) and 10.4 (phase 3b) in schools and 2.97 (phase 3a) and 2.77 (phase 3b) in households. During reduced attendance (phases 3b and 4), more secondary cases originated in other contact areas than in schools.

**Fig 5:**
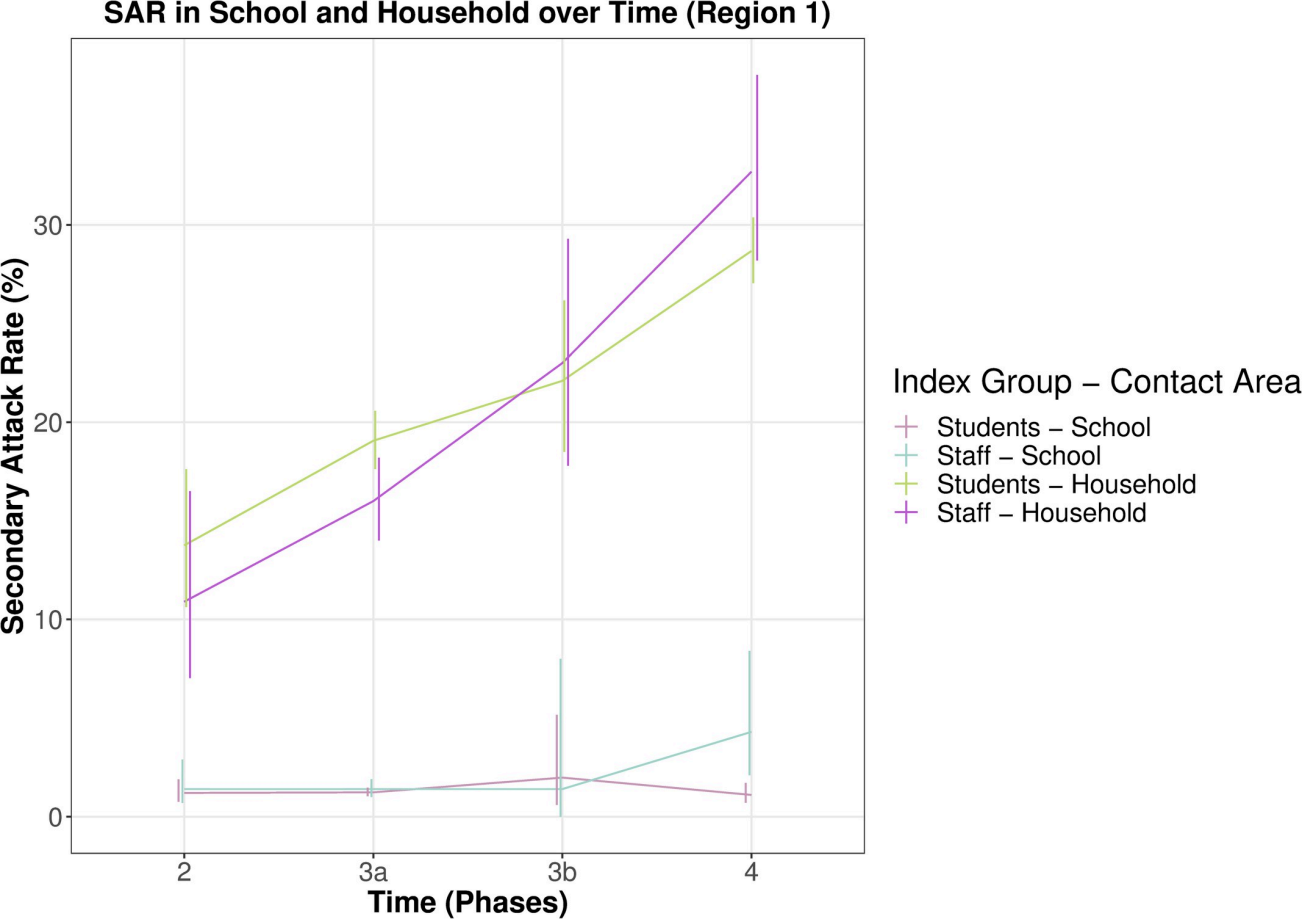
School and household SAR in students and staff over time. Comparison of school and household specific SARs for students and staff for Region 1. Vertical lines represent 95% CIs. Phase 2 summer plateau 2020, phase 3a second wave with open schools, phase 3b second wave with school closures, phase 4 mixed class models and VOC alpha. CI, confidence interval; SAR, secondary attack rate; VOC, variant of concern.

In phase 4 (VOC alpha), household SARs in students and staff were 1.5 to 2 times higher than phase 2 and phase 3a, whereas school specific SARs did not change significantly. School-specific SARs of staff members were higher for phase 4 at 4.3% (95% CI 2.1% to 8.4%) compared to 1.4% (95% CI 1% to 1.9%) in phase 3a and the average of 1.5% (95% CI 1.2% to 2%) (Fig 5).

Most secondary cases among school contacts of staff occurred in over 18-year-olds (57.7% or 30 out of 52) with a SAR of 4% (95% CI 2.8% to 5.7%), compared to 0.8% (95% CI 0.5% to 1.3%) in the under 10-year-olds and 1% (95% CI 0.4% to 2%) in the 10-to-18-year-olds.

### Effects of school-specific non-pharmaceutical measures on infection dynamics in schools (c)

Based on NUTS-3 level data reported by the KMK for W09-25/2021, a multiple regression model for the official active cases per 100,000 students and teachers found that in counties with a mask mandate during class in all types of schools (i.e., primary schools as well as secondary schools), the average number of 2-weekly cases was lower by 55.5 per 100,000 (95% CI 47.7 to 63.4, *p* < 0.001) and 55.6 (95% CI 39.6 to 71.6, *p* < 0.001) cases for students and teachers, respectively (Table 5). The adjustment for population incidence even after adjusting for the effect of measures showed an increase of student and teacher incidence with population incidence (0.3 and 0.5 per each increase of 1/100,000 per 14 days). The mask mandate in class for all types of schools was associated with a reduction of case numbers relative to the general population of 29.8% (95% CI 24.7% to 34.9%, *p* < 0.001) for students and 24.3% (95% CI 12.7% to 35.9%, *p* < 0.001) for teachers (Table 1 of Text E in S1 File). This result withstood sensitivity analysis of interaction with time periods of increasing infections with less measures or periods of decreasing infections and more measures implemented as well as to using a negative binomial model more appropriate for the non-normal distribution found in most variables (Tables 2 and 3 of Text E in S1 File).

**Table 5:** Regression results for active cases per 100k students or teachers, respectively, on the 2-week-lags of school-specific NPIs.

	Students		Teachers	
Variable	Increase or decrease in notified infections/100,000 persons/14 days (95% CI)	*p*-value	Increase or decrease in notified infections/100,000 persons/14 days (95% CI)	*p*-value
**2-week incidence per 100k inhabitants**	0.3 (0.28; 0.32)	<0.001	0.5 (0.47; 0.55)	<0.001
**Attendance in schools**				
Open schools	baseline		baseline	
School vacation	−8.8 (−19.3; 1.6)	0.098	−46.8 (−68.5; −25.2)	<0.001
School closures	−60.4 (−73.4; −47.7)	<0.001	−91.6 (−118.1; −65)	<0.001
Reduced presence in schools	−19.3 (−29.6; −9.1)	<0.001	−50.3 (−71.5; −29.1)	<0.001
**Mask mandates**				
No mask mandate or voluntary masking	baseline		baseline	
Partial mask mandate in some or all schools	−17.8 (−25.5; −10)	<0.001	9.9 (−5.2; 25.1)	0.199
Mandatory masks in all school classes	−55.5 (−63.4; −47.7)	<0.001	−55.6 (−71.6; −39.6)	<0.001
**Testing**				
No testing in schools	baseline		baseline	
Voluntary testing in schools	45 (38.2; 51.9)	<0.001	17.8 (4; 41.6)	0.011
Mandatory testing in schools	49.8 (41.2; 58.5)	<0.001	6.5 (−11.3; 24.3)	0.475
**Percentage of completely vaccinated population in corresponding federal state [cont.]**	−4.1 (−4.9; −3.2)	<0.001	−5.5 (−7.2; −3.8)	<0.001
**Urbanicity** †				
Rural	baseline		baseline	
Urban (RegioStar71/72)	21.3 (11.9; 30.7)	<0.001	−44.2 (−63; −25.5)	<0.001
**Deprivation Index** *				
Deprivation Index 0–0.5	baseline		baseline	
Deprivation Index 0.51–1	−7.8 (−16; 0.33)	0.06	81.5 (65.2; 97.7)	<0.001
R^2^ [as %] (sample size)	37.7 (3,809)		32.7 (3,979)	

See Supplementary Methods (Text B in S1 File). Attendance in schools: Open Schools = 81% to 100% of students in presence, School vacation = 0%, School closures 1% to 20%, Reduced presence in schools = 21% to 80%. Partial mask mandate in some or all schools: mask mandates only for secondary schools, for all schools but not in class, or in class only for secondary schools. The first row describes the coefficient estimates of the 2-week-incidence per 100k population of the regional population as a control variable.

*Deprivation Index: RKI German Index of Social Deprivation [31], where 0 is the lowest and 1 the highest level of social deprivation.

†Urbanicity Index by Federal Ministry of Transport and Infrastructure; 71 = metropolis, urban center defined by infrastructure and service availability in the region; 72 = regiopolis, regional urban centre defined by infrastructure, and service availability in region [32].

Lower attendance rates among students within regions were associated with reductions in infection risks of teachers and students in the OLS model. Vacation and reduced presence class models were associated with a greater reduction in the risk of infection for teachers than for students. In sensitivity analyses using a negative binomial distribution, an effect of reduced attendance was only visible up to calendar week 18 in both teachers and students with increasing incidence and prior to the *Bundesnotbremse* law (Text E in S1 File).

Higher vaccination coverage in the population was associated with a decrease in notified infections in students as well as teachers.

Mandatory testing in schools in our study period was associated with an average increase of 49.8 notifications per 100,000 per 14 days (95% CI 41.2 to 58.5, *p* < 0.001) among students.

Urban counties were associated with a higher number of notified infections in students and a lower number in teachers compared to rural counties. A high degree of social deprivation in a county was associated with higher numbers of infections in teachers (Text E in S1 File).

### Contribution of contacts in schools to overall transmission in the population (d)

**Fig 6:**
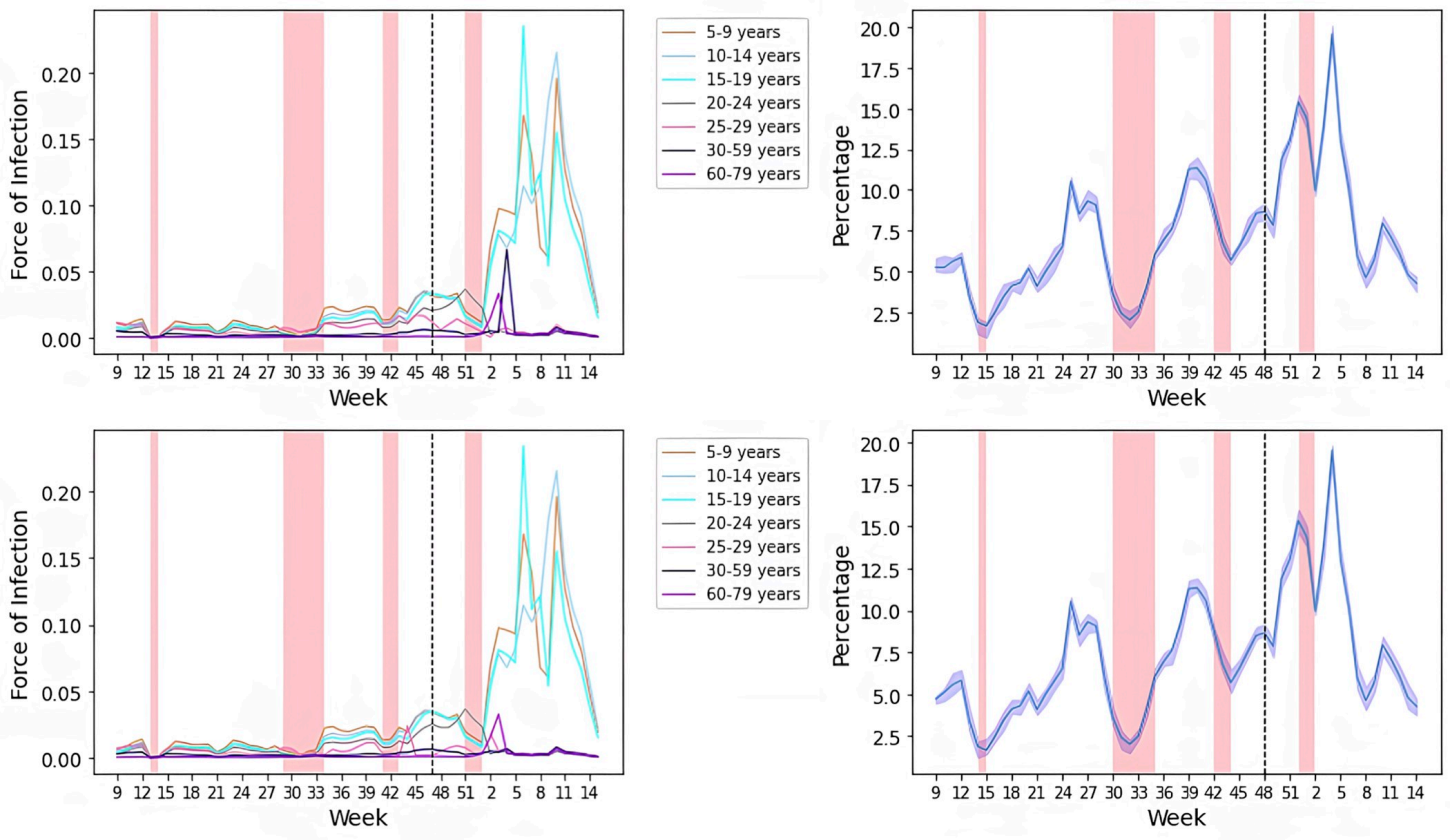
Estimated force of infection and contribution of contacts of infected persons in school to overall transmission in Germany week 9/2021 to 15/2022, accounting (c and d) and not accounting for underdetection (a and b). Pink areas represent periods when the majority (at least 7) federal states have school vacations. Vacation periods in federal states in Germany differ by multiple weeks and will span periods prior and post to the areas marked. W16-21 include county-specific incidence-dependent school closures which affected less than 50% of counties in Germany. The dotted line represents the onset of predominance of the delta variant among cases in Germany. Age-specific underestimation parameters were continued as for the delta variant, as no estimates for age-specific underdetection of the omicron variant were available at the time of publishing.

We used the NUTS-2 (federal state) level data by the KMK and RKI data from survstat for the compartmental model described in Fig 1. Not accounting for underestimation of infections by notifications, we found a high variability in the contribution of contacts from school infections to the overall transmission during W09-48/2021 (phase 4 with VOC alpha to phase 6 with VOC delta) from 2% to 12%. Accounting for age-specific underestimation of notified infections in comparison to actual infections based on seroprevalence estimates for alpha and delta variants, this range was 2% to 20% (Fig 6). The contribution was lowest during vacations or school closures. The peaks occurred with open schools. The W24/21 peak coincides with a 7-day average population incidence of 6 cases per 100,000 inhabitants and the W40/21 peaks occurs at 65 per 100,000 7-day population incidence. The peak at W12/21 occurs with reduced presence class models and a population incidence of 130 per 100,000 inhabitants (Fig A in S1 File).

We continued to use age-specified underdetection estimates for the delta variant after W48/2021 (omicron as predominant variant) for lack of data. We observed a peak in the contribution of schools to population infections reaching a maximum of 20% in W4/2022 (phase 7, VOC: omicron) at a population incidence of 1,337 per 100,000 inhabitants and a minimum of 4% in W14/2022 (phase 7) at 1,179 per 100,000 inhabitants.

## Discussion

This study presents findings for school-associated infection dynamics during the first 2 years of the SARS-CoV-2 pandemic in Germany with data covering pandemic phases with 4 variants (wild-type, alpha, delta, and omicron). We showed that the infection risk for students and teachers is higher than that of the general population during normal attendance periods and that mask mandates in all schools and during classes as well as reduced attendance models in particular were associated with reducing the risk of infection and transmission in schools. Transmission in schools occurred predominantly from staff to staff more than student to student, student to staff, and staff to student. Whereas most contacts of student or staff index cases were reported in schools, most secondary cases occurred in household contacts. The contribution of contacts in schools to population infections was as high as 20% during open schools and 2% to 3% during severely reduced attendance (vacations or closures with only emergency/holiday child care) in modelling estimates.

Infection risk for students and staff was higher than the population in all phases of normal attendance and equal or lower during reduced attendance. This effect seemed exacerbated, in particular for staff, during low levels of measures (phase 3a, wild-type) and mitigated most likely through tightened measures including priority vaccination, strict surgical masking, and reduced attendance models (phase 4, VOC: alpha). This is in line with findings from the UK [33,34], where prevalence in school-aged children was higher than or similar to the adult population in late 2020 and similar or lower after school closures in early 2021. Similarly, seroprevalence studies reported varying outcomes for the risk of infection of students relative to adults depending on their timing and approach of investigation [35–37] and were mostly limited to periods before the delta variant was introduced. For teachers, an association with a higher risk of infection in times of higher community incidence and with open schools was described [16].

Whereas increased age of students was associated with a higher risk of infection, as previously shown [17,19], this effect appeared reduced with the alpha variant (phase 4) and is likely to have reduced further with even more transmissible variants [15].

This study adds to previous findings by extending the observation of student and staff risk of infection into the omicron era. During phase 6 (VOC: delta) and phase 7 (VOC: omicron), the effect of stringent classroom measures while maintaining full attendance appeared insufficient to reduce infection risk for both staff and students to population levels. In particular, the difference in vaccination coverage between the adult and student population could have exacerbated student infection risk relative to the population and staff during phase 6 (delta) and 7 (omicron). Early findings from the US support this idea, showing a greater protection in vaccinated students [38]. Whereas teachers as a vocational group were vaccinated with priority in early 2021, vaccination for students aged 12 to 17 in Germany started in W33/2021, and for 5-to-11-year-olds in W49/2021, with a recommendation only for those 5 to 11 year olds with comorbidities. Previous research showed no benefits for students by high vaccination rates in teachers as well as a required minimum of 55% to 60% student vaccination coverage to see benefits of other measures decrease [39], levels not reached in Germany during the study interval. On the other hand, in our multiple regression analysis of NUTS-3 data, we do find a clear indication of lower infection risks in students and teachers in those regions with higher vaccination coverage even for vaccination coverages below 20% in early 2021. More than a third of students were notified of having been infected during the omicron wave in Germany (phase 7) in some federal states. The risk of teachers was higher than the general population most likely due to their exposure to this susceptible, high-incidence group.

In this study, we demonstrated that student and staff SARs can be divided into a school setting with many contacts but a lower risk of transmission and adjustable measures and households with fewer contacts and a higher risk of transmission as a space largely void of measures. This was confirmed for schools by daily contact testing studies [21], however, not in relation to households and other contact areas at the same time. Other contact areas played a consistent role, contributing more secondary cases than schools during periods of reduced attendance. Stricter measures and reduced attendance models were associated with a stabilisation of in-school SARs at the transition from wild type (phase 3a) to more transmissible alpha-variant predominance (phase 4), while household SARs more than doubled. Most secondary cases of both students and staff occurred in households. Reported SARs for students in households were similar to previous findings [40,41], with most studies emphasising a higher household SAR for adults than children, with notable exceptions [42], and a lower risk of child-to-adult transmission than vice versa. Household SAR for adults was in part higher than found for staff in our study [40,41]. This can be due to differences in contact tracing between retrospective, passive surveillance data, and prospective investigative contact tracing and testing. In schools, transmission from staff to staff was more likely than student to staff. This was in accordance with previous findings, most concluding that adult-to-adult and adult-to-child were the most common transmission routes in schools [16,42–44] and our finding that transmission risk was associated both with higher age of the of index cases as well as contacts.

In our analysis, regions with NPIs such as strict mask mandates (surgical masks in all schools during class) and reduced presence models had lower infection and transmission risks in teachers and students in absolute terms and relative to the population. We estimated a reduction by 24% to 30% of infection risk for both students and teachers relative to the population in counties implementing mandatory surgical mask wearing during class in all schools. Previous studies estimated this relative reduction at 23% [45] and 37% [46].

Furthermore, all interventions reducing attendance rate of students (rotating and distance classes as well as school closures and vacations) were associated with reducing the risk of infection of students and teachers. This effect was not as clear in the sensitivity analyses using a negative binomial regression, where an effect of partial or full school closure was mainly found during weeks of increasing incidence in Germany, possibly due to the association with the *Bundesnotbremse* law forcing closures in those regions with high incidences. In principle, an association between reduced attendance and reduced infection rates was confirmed by 12 out of 13 studies in a recent review [23]. Concurrently, a higher risk of infection for students and teachers was associated with higher deprivation indices. This was known for Germany, though not specifically for schools [47,48], and is reported for other countries [35,49].

A protective effect was associated with higher vaccination rates in the population. We found a higher number of notified cases among students with mandatory testing, likely through reducing underdetection. Studies showed a clear benefit for the regular (asymptomatic) testing as it was conducted in German schools [39]. Interestingly, in sensitivity analyses (Text E in S1 File), we found a decrease in infection risk of primary school students when attendance in school increased, whereas attendance is positively correlated with infection risk for all other student and staff groups. Reasons could include stricter adherence to rules while in schools and less time spent in households and other contact areas with a higher adult-to-child transmission. One study describes differential mask adherence of primary and secondary school students [50], however, without reporting confidence intervals or clinical significance.

In dynamic modelling based on NUTS-2 KMK data in Germany, we estimated that the contribution of infection dynamics in schools to that of the general population was up to 20%. Peaks occurred during normal attendance and under strict hygiene and testing measures. Whereas during alpha and delta variant waves the maximum contribution was 13%, the 20% peak early during the omicron wave is likely due to the large gap in vaccine coverage between adults and school-aged children, in particular 5-to-11-year-olds. Modelling studies have suggested that lack of vaccine coverage was the main contributor to higher incidences in the young population [39] and that this brings an increased risk for households with school-age children [51].

During school vacations and closures, we estimated a contribution of school contacts to population infections as low as 2%. The highest contribution during reduced attendance class models was at 7%. In principle, our findings are in line with previous modelling based on highly heterogenous ecological data for the first and second wave in Europe estimating that school closures resulted in >30% and <15% reductions of transmission, respectively [8,52].

Limitations of our work are inherent in the notification process to both public health and educational agencies as well as the gathering of aggregate county-specific data (Text F in S1 File). Both notification data itself as well as contact data is an underestimate of the actual infection dynamic. Which populations are more and less affected by underdetection changes over time. The advent of regular testing in schools could have led to a relatively lower underdetection among students and inflated risk of infection relative to the population. However, concurrently with schools, regular testing was at times required in other domains of society, such as in workplaces, venues, social gatherings, and public transport. The regular testing in schools, in particular during the delta and omicron waves, took place in an environment of ubiquitous testing mandates across societal domains, for both vaccinated and unvaccinated persons depending on local government policy and changing over time. Testing mandates alone would thus not account for the disparity in risk of infection observed.

For the alpha and delta variants, we used age-specific estimates of underdetection from seroprevalence studies to account for this in our analysis of the contribution of schools to population infections. Including age-specific underdetection ratios did not significantly change the outcome for time periods of alpha and delta variant predominance and mandatory regular in-school testing. As of now, no estimates for the omicron variant were available. Furthermore, the contact matrices and transmission parameters specific to age group and contact area and their scaling in response to policy changes underlie confounders of the original research they were adapted from. The contribution of schools to the pandemic is thus subject to unquantified biases hidden in transition parameters adapted in particular for the omicron phase.

SARs are limited by several factors. Contact tracing is inherently limited by both interviewer capacity and ability and interviewee memory and honesty, however, with schools and households as contained domains the error margin is limited. Among contacts, the data did not indicate whether these were still susceptible to infection. However, it can be assumed that biases are similar in each region as they are situated within the same country and timeline of events. All considered, the SARs presented here described further transmission after cases have been detected in the school with limitations.

Residual confounding in the regression analysis is possible as we use aggregate county or school measures and did not have access to individual-level confounding factors, e.g., on distribution of parental professions or industrial make-up of the counties included. We tried to include the most important confounding factors such as infection dynamics in the local population as well as deprivation and urbanicity of counties in the analysis. Furthermore, population-level NPIs are not directly included in the analysis. Across the observation period in the 275 NUTS-3 regions, the number of individual NPI changes and high dimensionality and multicollinearity of measures applied and withdrawn in clusters prohibited their use in this analysis until more reliable parameters on effect and correlation of individual population-level NPIs are known. We attempted to include the effects of the other NPIs indirectly by using the population incidence as a control variable and reporting the relative effect between the student and teacher populations directly affected by school-based and population-based interventions and the general population affected directly by general NPIs and only indirectly by school-based interventions.

Future research should focus on population-level trials of hygiene measures and testing policies to help us better understand the exact effect of these measures on infection and non-infection-related endpoints. Platforms for their quick roll-out and implementation should be established interpandemically to not be reliant on only retrospectively collected data from different sources in the next epidemic or pandemic.

In this study, we could show that students and staff had a higher risk of infection relative to the population as schools continued normal presence classes during a pandemic environment. In Germany, school-related NPIs, in particular, masking and reduced attendance, were associated with mitigating the spread of the virus among both students and teachers. Contacts in schools were a relevant contributor to population transmission during phases with open schools, in particular, during the omicron wave.

## Supporting information

S1 STROBE Checklist STROBE Checklist. (PDF)

S1 File Supplementary files. Text A. Data sources and data collection overview. Text B. Additional methodology. Fig A. Overview of incidences across RKI phases of the pandemic. Table A. Infection control measures for schools in Region 1. Table B. Infection control measures for schools in Region 2. Table C. Infection control measures for schools in Region 3. Table D. Infection control measures for schools in Region 4. Table E. Infection control measures for schools in Region 5. Fig B. Incidences of population in 5 regions providing regional data. Fig C. Incidences of population, students, and staff in Region 1. Fig D. Incidences of population, students, staff and teachers in Region 2. Fig E. Incidences of population and students in Region 3. Fig F. Incidences of population and students in Region 4. Fig G. Incidences of population and students in Region 5. Fig H. Incidences of students in Regions 1–5. Fig I. Incidences of staff in Regions 1 and 2. Text C. Additional results 1: Infection risk. Table F. Infection risk across regions and subgroups (local agency regional data). Table G. Infection risk, crude risk ratios, and quarantine risk across federal states (KMK data). Table H. Secondary attack rates in students by region, phase, and age (local agency regional data). Text D. Additional results 2: Secondary attack rates. Text E. Additional results 3: Effects of NPIs in schools. Text F. Additional limitations. Text G. Supplement references.
(DOCX)

S1 Study Protocol Study Protocol. (DOCX)

## Abbreviations

CI: confidence interval; COVID-19, Coronavirus Disease 2019
CRR: crude risk ratio
ICU: intensive care unit
NPI: nonpharmaceutical intervention
RKI: Robert Koch Institute
SAR: secondary attack rate
SARS-CoV-2: Severe Acute Respiratory Syndrome Coronavirus 2
VOC: variant of concern
