# Supplementary material for: Infection and transmission risks of COVID-19 in schools and their contribution to population infections in Germany: A retrospective observational study using nationwide and regional health and education agency notification data
Source: PLoS Med. 2022 Dec 20;19(12):e1003913. doi: 10.1371/journal.pmed.1003913 (PMC9767368; doi:10.1371/journal.pmed.1003913)
Supplement: S1 STROBE Checklist — (PDF) [file pmed.1003913.s001.pdf]

STROBE Statement—checklist of items that should be included in reports of observational studies

	Item No.	Recommendation	Section	Relevant text from manuscript
**Title and abstract**	1	(*a*) Indicate the study's design with a commonly used term in the title or the abstract	Title	retrospective observational study
		(*b*) Provide in the abstract an informative and balanced summary of what was done and what was found	Abstract	We used German federal state (NUTS-2) and county (NUTS-3) data from national and regional public health and education agencies to assess infection risk and secondary attack rates (SARs) from March 2020 to October 2021 in Germany. We used multiple regression analysis and infection dynamic modelling, accounting for urbanity, socioeconomic factors, local population infection dynamics and age-specific underdetection to investigate the effects of infection control measures.<br>In counties with mandatory surgical mask wearing during class in all schools infection risk of students and teachers was reduced by 56/100.000 persons per 14 days and by 30% and 24% relative to the population respectively. Overall contribution to population infections of contacts in school settings was 2-13%. It was lowest during school closures and vacation and highest during normal presence class intervals. Infection risk for students increased with age and was similar to or lower than the population risk during second and third waves in Germany and higher in summer 2021. Infection risk of teachers was higher than the population during the second wave and similar or lower thereafter with stricter measures in place. SARs for students and staff were below 5% in schools throughout the study period. SARs in households more than doubled from 14% W21-39/2020 to 29-33% in W08-23/2021. Most contacts were reported for schools, yet most secondary cases originated in households. In schools, staff predominantly infected staff and students predominantly infected students.

**Introduction**				
Background/rationale	2	Explain the scientific background and rationale for the investigation being reported	Introduction	Schools have been a key target domain for nonpharmaceutical interventions (NPIs) in the SARS-CoV-2 pandemic. However, infection dynamics in schools, their impact on the wider population and the effect of NPIs remain ill-defined.<br><br>In Germany and Europe, studies on school transmission risk and contribution to overall transmission in the population are based on geographically and temporally limited data<br><br>Studies investigating school setting transmission in Germany are limited to small geographic units and short time periods.<br><br>In this study we obtained federal state and county level data for school settings as well as detailed regional data obtained from individual public health and educational agencies to assess infection risk, secondary attack rates, the contribution of schools to the overall transmission as well as the effect of NPIs in schools during the first 18 months of the pandemic in Germany.
Objectives	3	State specific objectives, including any prespecified hypotheses	Introduction,<br>S3 Text. 3.1 Aims	In this study we obtained federal state and county level data for school settings as well as detailed regional data obtained from individual public health and educational agencies to assess infection risk, secondary attack rates, the contribution of schools to the overall transmission as well as the effect of NPIs in schools during the first 18 months of the pandemic in Germany.<br><br>The primary aim of this study is a better understanding of the risk of infection, the transmission of SARS-CoV-2 in schools, and the contribution of in-school contacts to overall cases in the population over different phases of the pandemic in Germany.

				Moreover, we assess the reduction of risks of infection and transmission associated with specific measures taken in schools.<br>Specific aims of the study are an analysis of the<br>1) risk of infection in students and staff in schools,<br>2) risk of notified infection for a contact person (*secondary attack rate*) of a notified student and staff index cases, and<br>3) effect of measures taken in schools to reduce the transmission of SARS-CoV-2.<br>Whereas health and education agency data were used for Aims 1) and 2), data of the Standing Conference of Ministers of Education and Cultural Affairs in Germany (*KMK*) at NUTS-3 level was used for aim 3).
**Methods**				
Study design	4	Present key elements of study design early in the paper	Methodology – study design and data sources	we performed a retrospective observational study of prospectively collected SARS-CoV-2 infection case report data from health and educational authorities in Germany
Setting	5	Describe the setting, locations, and relevant dates, including periods of recruitment, exposure, follow-up, and data collection	Methodology – study design and data sources, S1, S2, S3	Methodology:<br>we used data collected by the Standing Conference of Ministers of Education and Cultural Affairs in Germany (KMK) (23). Federal state agencies collected data weekly in a structured format from all schools within their jurisdiction and included the number of students, classes and staff infected or absent due to quarantine and school closures. Federal state-level data is publicly available from calendar week (W) 46/2020 onwards, excluding school closures. In addition, we received unpublished county-level data, collected in the same manner on the same parameters

				S1 Table. Data Sources. *Table 1 Data obtained from public health agencies on SARS-CoV-2 infections in students and teachers and their contacts. Region 1. Region 2. Region 3. Region 4. Region 5*<br><br>S2 Table. Overview of Regional Data Collection. *Region 1. Region 2. Region 3. Region 4. Region 5*<br><br>S3 Text. Additional Methodology. 3.2 Health/education agency data. 3.3 Data of the KMK<br>Items included for analysis were the date of case notification, age of the index case, contacts and secondary cases (R1, R3-5), contact age (R1, R3, R5) and contact area (R1). Data was requested from February 2020 to March 2021 and was provided as available at the time of data sharing. The data was cleaned, and discrepancies and questions cleared with the providing agency.<br><br>**Texts from Supplement not copied in due to excessive length**
Participants	6	(*a*) *Cohort study*—Give the eligibility criteria, and the sources and methods of selection of participants. Describe methods of follow-up<br>*Case-control study*—Give the eligibility criteria, and the sources and methods of case ascertainment and control selection. Give the rationale for the choice of cases and controls<br>*Cross-sectional study*—Give the eligibility criteria, and the sources and methods of selection of participants	Methodology – study design and data sources	Public health authorities report case data according to the Infection Protection Act (IfSG). Cases were defined by direct viral detection via nasopharyngeal swabs using PCR or by cultural isolation of the pathogen.<br><br>prospectively collected SARS-CoV-2 infection case report data from health and educational authorities in Germany from 5 regions<br><br>data collected by the Standing Conference of Ministers of Education and Cultural Affairs in Germany (KMK). Federal state agencies collected data weekly in a structured format from all

				schools within their jurisdiction and included the number of students, classes and staff infected or absent due to quarantine and school closures.<br><br>we sourced population infection and incidence data from the Robert Koch Institute (RKI) SURVSTAT tool, described as incidence per 100000 inhabitants
		(*b*) *Cohort study*—For matched studies, give matching criteria and number of exposed and unexposed<br>*Case-control study*—For matched studies, give matching criteria and the number of controls per case		
Variables	7	Clearly define all outcomes, exposures, predictors, potential confounders, and effect modifiers. Give diagnostic criteria, if applicable	Methodology – study design and data sources; Methodology – data analysis	Cases were defined by direct viral detection via nasopharyngeal swabs using PCR or by cultural isolation of the pathogen.<br><br>We performed a descriptive analysis of infection risk for students and teachers as cumulative risks and crude risk ratios for specified periods of time with 95% Confidence Intervals (CI). We calculated secondary attack rates (SAR) as percentages with 95% CI of those being identified as notified infections in all known contacts.<br><br>We investigated infection control measures and other factors influencing infection risk of teachers or students in a multiple linear regression model. We used an ordinary least squares (OLS) model to test for statistical associations of the official active cases per 100000 students and teachers as reported by the KMK for the county level on the two-week-lags of the attendance rates (by educational level), the stringency of mask mandates (Table 1 in S3 Text) and the mandate and stringency of testing in schools. We controlled for the two-week incidence per 100000 inhabitants of the corresponding county, the percentage of fully vaccinated persons in the corresponding federal state, the socioeconomic

status and geography of the county (S3 Text, S11 Text). Furthermore, we tested whether the school specific NPIs had an over proportional effect on the school population compared to the overall population. We proceeded similarly to the first approach, yet did not include a control group as an explanatory variable but instead divided the active cases among the students or teachers, respectively, by the 14-day incidence of the total population of the district. This way we estimated the effect of the NPIs on cases in schools relative to cases in the population, thus estimating whether the NPIs in schools have a significantly different effect on the overall population as on the school population.

The infection dynamics in schools were estimated using a SEIRS (Susceptible-Exposed-Infectious-Recovered-Susceptible) model previously described(27). This distinguishes between healthy but susceptible individuals, those infected but not yet infectious (exposed), and symptomatic and asymptomatic patients. In addition, we included compartments for hospitalisations, patients entering intensive care units (ICUs) and persons with long-COVID, i.e. those who continue to have sequelae after recovery. In the final state, the patients are recovered or dead. Furthermore, we assume a reinfection process. We split the recovery compartment into a compartment for those recovered from COVID and a long-COVID compartment, since we assume that both have a different reinfection rate.
The remaining parameters represent time periods of transitions between the different statuses as well as probabilities for the transitions and are estimated from international data and literature research. The data for the model is composed of reports from KMK(23), RKI (25) and the DIVI Intensive Care Register(28). We accounted for age-specific underdetection taking pandemic

				period- and age-specific underdetection ratios from a large seroprevalence study in Germany(29).
Data sources/ measurement	8*	For each variable of interest, give sources of data and details of methods of assessment (measurement). Describe comparability of assessment methods if there is more than one group	Methodology – study design and data sources; S2 Table	We performed a retrospective observational study of prospectively collected SARS-CoV-2 infection case report data from health and educational authorities in Germany<br>Public health authorities report case data according to the Infection Protection Act (IfSG). Cases were defined by direct viral detection via nasopharyngeal swabs using PCR or by cultural isolation of the pathogen.<br><br>S2 Table with information on regional agencies:<br>R1, R3, R4, R5:<br>All patients with detection of SARS-CoV-2 were contacted and interviewed by the local health department.<br><br>R2:<br>The condition for a case was the student or teacher self-reporting a positive SARS-CoV-2 PCR test to the school. The list of cases includes all infections reported by the school to the education authority.<br><br>Secondly, we used data collected by the Standing Conference of Ministers of Education and Cultural Affairs in Germany (KMK) (23). Federal state agencies collected data weekly in a structured format from all schools within their jurisdiction and included the number of students, classes and staff infected or absent due to quarantine and school closures.

				Thirdly, we sourced population infection and incidence data from the Robert Koch Institute (RKI) SURVSTAT tool, described as incidence per 100000 inhabitants.
Bias	9	Describe any efforts to address potential sources of bias	Methodology – Data analysis; Discussion; S12 Text. Additional Limitations	We controlled for the two-week incidence per 100000 inhabitants of the corresponding county, the percentage of fully vaccinated persons in the corresponding federal state, the socioeconomic status and geography of the county (S3 Text, S11 Text). Furthermore, we tested whether the school specific NPIs had an over proportional effect on the school population compared to the overall population.<br><br>Data was analysed per calendar week (W) as the main time unit in German state agency publications and avoids the weekly seasonality of data reporting (4) (no school on weekends, some local health agencies in Germany do not report data on weekends or Sunday).<br><br>We accounted for age-specific underdetection taking pandemic period- and age-specific underdetection ratios from a large seroprevalence study in Germany<br><br>Limitations of our work are inherent in the notification process to both public health and educational agencies as well as the gathering of aggregate county-specific data (S12 Text). Both notification data itself as well as contact data is an underestimate of the actual infection dynamic. We attempted to account for underdetection with age-specific estimates taken from seroprevalence studies. This could make our estimate of contribution of school-contacts to overall transmission a potential underestimate. Residual confounding in the regression analysis is a possibility as we use aggregate county or school measures and did not have full access to individual-level confounding factors,

				e.g., on distribution of parental professions or industrial make-up of the counties included. We tried to include the most important confounding factors on infection dynamics in the population as well as deprivation and urbanity of counties in the analysis. SARs are limited by several factors. Contact tracing is inherently limited by both interviewer capacity and ability and interviewee memory and honesty, however with schools and households as contained domains the error margin is limited. Among contacts, the data did not indicate whether these were still susceptible to infection. However, it can be assumed that biases are similar in each region as they are situated within the same country and timeline of events. A reduction in the proportion of the susceptible population through vaccination or infection is assumed relevant from March 2021, where it is included as a parameter in regression analysis. All considered, the SARs presented here described further transmission after cases have been detected in the school with limitations.<br><br>S12 Text. Additional Limitations. **Text not copied in due to excessive length**
Study size	10	Explain how the study size was arrived at	Methodology – study design and data sources; S2 Table	We performed a retrospective observational study of prospectively collected SARS-CoV-2 infection case report data from health and educational authorities in Germany<br>Public health authorities report case data according to the Infection Protection Act (IfSG). Cases were defined by direct viral detection via nasopharyngeal swabs using PCR or by cultural isolation of the pathogen.<br><br>All patients with detection of SARS-CoV-2 were contacted and interviewed by the local health department.

	Federal state agencies collected data weekly in a structured format from all schools within their jurisdiction and included the number of students, classes and staff infected or absent due to quarantine and school closures.

Continued on next page

Quantitative variables	11	Explain how quantitative variables were handled in the analyses. If applicable, describe which groupings were chosen and why	S3 Text. Additional Methodology; S11 Text. Additional Results	Supplementary Results 3, Table 4 and 5 show previous analyses using the variable coding in Table 6. The original (full) model is presented next to the final model. The original selection of variables yielded some insensible and insignificant estimates due to multicollinearity (Table 7), thus some variables were excluded or rearranged for further analysis in the final model and the new variable grouping as per S3 Text, Table 1.
Statistical methods	12	(*a*) Describe all statistical methods, including those used to control for confounding	Methodology – data analysis	We performed a descriptive analysis of infection risk for students and teachers as cumulative risks and crude risk ratios for specified periods of time with 95% Confidence Intervals (CI). We calculated secondary attack rates (SAR) as percentages with 95% CI of those being identified as notified infections in all known contacts. Confidence intervals for binomial values were calculated using the Agresti-Coull<br><br>We investigated infection control measures and other factors influencing infection risk of teachers or students in a multiple linear regression model. We used an ordinary least squares (OLS) model to test for statistical associations of the official active cases per 100000 students and teachers as reported by the KMK for the county level on the two-week-lags of the attendance rates (by educational level), the stringency of mask mandates (Table 1 in S3 Text) and the mandate and stringency of testing in schools. We controlled for the two-week incidence per 100000 inhabitants of the corresponding county, the percentage of fully vaccinated persons in the corresponding federal state, the socioeconomic status and geography of the county (S3 Text, S11 Text). Furthermore, we tested whether the school specific NPIs had an over proportional effect on the school population compared to the overall population. We proceeded similarly to the first approach, yet did not include a control group as an explanatory variable but

instead divided the active cases among the students or teachers, respectively, by the 14-day incidence of the total population of the district. This way we estimated the effect of the NPIs on cases in schools relative to cases in the population, thus estimating whether the NPIs in schools have a significantly different effect on the overall population as on the school population.
To control for the overall infection activity during that period, we downloaded weekly incidences per 100000 inhabitants for the districts for the total population of said districts (3). To account for socioeconomic effects, we used an index derived by Kroll (9). The current values this index for the year 2015 were provided by Kroll (10). Moreover, we accounted for potential geographical effects of the districts, i.e. we may witness higher ceteris paribus (c.p.) infection risks for students who are living in highly rural areas and need to commute to school using public transports, which could increase their risk of infection. To test these effects on infection risks of students and teachers, we discriminated all districts according to the so-called RegioStar7 categorisation by the Federal Ministry of Transport and Digital Infrastructure, which defines 7 different categories of LAU regions (11).

The infection dynamics in schools were estimated using a SEIRS (Susceptible-Exposed-Infectious-Recovered-Susceptible) model previously described(27). This distinguishes between healthy but susceptible individuals, those infected but not yet infectious (exposed), and symptomatic and asymptomatic patients. In addition, we included compartments for hospitalisations, patients entering intensive care units (ICUs) and persons with long-COVID, i.e. those who continue to have sequelae after recovery. In the final state, the patients are recovered or dead. Furthermore, we assume a reinfection process. We split the recovery compartment into a compartment for those recovered from

			COVID and a long-COVID compartment, since we assume that both have a different reinfection rate. The remaining parameters represent time periods of transitions between the different statuses as well as probabilities for the transitions and are estimated from international data and literature research. The data for the model is composed of reports from KMK(23), RKI (25) and the DIVI Intensive Care Register(28). We accounted for age-specific underdetection taking pandemic period- and age-specific underdetection ratios from a large seroprevalence study in Germany(29).
	(*b*) Describe any methods used to examine subgroups and interactions	Methodology – data analysis; Results; S1; S7; S8; S9; S10; S11 Text. Additional Results	S1 Table gives the overview of categories of data available, including a description by region.<br><br>Items included for analysis were the date of case notification, age of the index case, contacts and secondary cases (R1, R3-5), contact age (R1, R3, R5) and contact area (R1).<br>Secondary attack rates (SARs) as percentages were calculated where possible.<br><br>Student and staff infection risk and incidences were calculated with school population data from regional or federal state authorities, analysed by age and school form as percentages where possible and described.<br><br>Results Table 2<br>S7 Tables: Infection Risks Across Regions and Subgroups<br>S8 Text. Additional Results 1: Infection Risk<br>S10 Text. Additional Results 2: Secondary Attack Rates<br><br>Supplementary Results 3, Table 2 and 3 show the results of a sensitivity analysis splitting the observation period into rising

			infections during the 4th wave (W08-16) and waning infections during the 4th wave (W17-23).<br><br>We accounted for age-specific underdetection taking pandemic period- and age-specific underdetection ratios from a large seroprevalence study in Germany
	(*c*) Explain how missing data were addressed	Results; S1 Tables; S2 Tables	Both notification data itself as well as contact data is an underestimate of the actual infection dynamic. We attempted to account for underdetection with age-specific estimates taken from seroprevalence studies. This could make our estimate of contribution of school-contacts to overall transmission a potential underestimate. Residual confounding in the regression analysis is a possibility as we use aggregate county or school measures and did not have full access to individual-level confounding factors, e.g., on distribution of parental professions or industrial make-up of the counties included. We tried to include the most important confounding factors on infection dynamics in the population as well as deprivation and urbanity of counties in the analysis. SARs are limited by several factors. Contact tracing is inherently limited by both interviewer capacity and ability and interviewee memory and honesty, however with schools and households as contained domains the error margin is limited. Among contacts, the data did not indicate whether these were still susceptible to infection. However, it can be assumed that biases are similar in each region as they are situated within the same country and timeline of events. A reduction in the proportion of the susceptible population through vaccination or infection is assumed relevant from March 2021, where it is included as a parameter in regression analysis. All considered, the SARs presented here described further transmission after cases have been detected in the school with limitations.

		S2 Table. Overview of Regional Data Collection – gives the data collector's perspective on issues with data completeness
(*d*) *Cohort study*—If applicable, explain how loss to follow-up was addressed<br>*Case-control study*—If applicable, explain how matching of cases and controls was addressed<br>*Cross-sectional study*—If applicable, describe analytical methods taking account of sampling strategy	S3 Text. Additional Methodology	Of around 400 local health agencies in Germany, 20% were contacted since March 2021, of which 7 agreed to provide their school-specific data, of which 5 were provided in time for analysis.<br><br>We received weekly data on known infections among students and teachers on the NUTS-3 level (Kreise or Districts) by the KMK for 14 of the 16 German federal state (except Bavaria and Hesse; Berlin and Hamburg are both counties and federal states).<br><br>To control for the overall infection activity during that period, we downloaded weekly incidences per 100000 inhabitants for the districts for the total population of said districts (3). To account for socioeconomic effects, we used an index derived by Kroll (9). The current values this index for the year 2015 were provided by Kroll (10). Moreover, we accounted for potential geographical effects of the districts, i.e. we may witness higher ceteris paribus (c.p.) infection risks for students who are living in highly rural areas and need to commute to school using public transports, which could increase their risk of infection. To test these effects on infection risks of students and teachers, we discriminated all districts according to the so-called RegioStar7 categorisation by the Federal Ministry of Transport and Digital Infrastructure, which defines 7 different categories of LAU regions (11).
(*e*) Describe any sensitivity analyses	S11 Text	Sensitivity Analysis<br>S11 Text, Table 2 and 3 show the results of a sensitivity analysis splitting the observation period into rising infections during the

				4th wave (W08-16) and waning infections during the 4th wave (W17-23).<br><br>S11 Text, Table 4 and 5 show previous analyses using the variable coding in Table 6. The original (full) model is presented next to the final model. The original selection of variables yielded some insensible and insignificant estimates due to multicollinearity (Table 7), thus some variables were excluded or rearranged for further analysis in the final model and the new variable grouping as per S3 Text, Table 1.
**Results**				
Participants	13*	(a) Report numbers of individuals at each stage of study—eg numbers potentially eligible, examined for eligibility, confirmed eligible, included in the study, completing follow-up, and analysed	S1 Table	S1 Table
		(b) Give reasons for non-participation at each stage	Results	Limitations of our work are inherent in the notification process to both public health and educational agencies as well as the gathering of aggregate county-specific data (S12 Text). Both notification data itself as well as contact data is an underestimate of the actual infection dynamic. We attempted to account for underdetection with age-specific estimates taken from seroprevalence studies.
		(c) Consider use of a flow diagram		
Descriptive data	14*	(a) Give characteristics of study participants (eg demographic, clinical, social) and information on exposures and potential confounders		S1 Table<br>S2 Table
		(b) Indicate number of participants with missing data for each variable of interest		Limitations to the data are inherent in the process of disease reporting and contact tracing. Underdetection of cases, especially in the younger age group, will affect infection risk. Regional differences in infection environment and NPI adherence affect infection risk and make inter-regional comparison difficult. NPIs

affecting domains of life other than schools were not considered and differed by federal state in Germany. R1, R2 and R4 collated data on students separately, with a margin of error in picking up the relevant classification of cases from contact tracing. R3 and R5 identified students based on age, thus older (in particular vocational) students will not be perfectly reflected in the analysis. Furthermore, health agencies collate cases based on registered place of residence. In particular R4 data will be affected by cross-border (country and county) commuters and administrative differentiation of cases by residence, as would be R1, R2 and R5 through peri-urban commuting.

Contact tracing fidelity depends on capacity and capability of health agencies, reporting by index cases and is inherently flawed. This is obvious in that a) the reproduction numbers for regions where full population datasets were provided (R3, R5) is below 1 and thus not compatible with the pandemic progression witnessed and b) on average, only 65% of index cases had any contacts reported at all (R1 54%, R3 54%, R4 42% and R5 84%), leading to a skewed picture of contacts and secondary attack rates as well as biases as to which index cases would have reported contacts and how or whether they would have been identified as secondary cases by authorities in due course. Furthermore, especially for household cases the direction of transmission in instances of multiple household cases cannot always be clearly ascertained, the same holds true for attribution of origin of infected to any one contact, particularly during phases of high transmission.

S9 Tables gives the number of cases with contacts reported. It is not known whether these were cases without contacts, without a detailed contact tracing or whether the index case did not report contacts.

		(c) *Cohort study*—Summarise follow-up time (eg, average and total amount)		S1 Table
Outcome data	15*	*Cohort study*—Report numbers of outcome events or summary measures over time	Abstract<br>Results<br>S1 Table<br>S6 Figures	We included (1) nation-wide NUTS-2 level data from calendar weeks (W) 46-50/2020 and W08-40/2021 with 304676 infections in students and 32992 in teachers; (2) NUTS-3 level data from W09-25/2021 with 85788 student and 9427 teacher infections and (3) detailed data from 5 regions covering W09/2020 to W27/2021 with 12814 infections, 43238 contacts and 4165 secondary cases for students (for teachers 14801, 5893 and 472 respectively).<br><br>Data obtained from KMK was collected on the federal state level for W46-50/2020 and W08-40/2021 (at time of analysis), covering 304676 student and 32992 teacher cases.<br><br>Data obtained from local authorities covered a total general population of 3970903, with 545409 (13.7%) students and 60401 (1.5%) teaching and other staff. We included 15433 index cases, 49131 contacts and 4637 secondary cases reported during the school year 2020/21 across 5 regions of Germany.<br><br>S1 Table<br>S6 Figures
		Case-control study—Report numbers in each exposure category, or summary measures of exposure		
		Cross-sectional study—Report numbers of outcome events or summary measures		
Main results	16	(*a*) Give unadjusted estimates and, if applicable, confounder-adjusted estimates and their precision (eg, 95% confidence interval). Make clear which confounders were adjusted for and why they were included	Results	Cumulative risk of notified SARS-CoV-2 infection for students was 0.96% W46-50/2020, 1.22% in phase 4, 1.16% in phase 5 and for teachers 1.77%, 1.53% and 0.47% respectively. At the

county level in W09-25, infection risk was 1.68% for students and 1.51% for teachers (Table 2, Table 1 in S7 Tables).

Infection risk in phases 2-4 ranged from 2-7.6% for the general population, 1.3-5.8% for students and 2.4-3.2% for staff (Table 2).

Overall – irrespective of contact setting - risk of infection after contact with a notified case (SAR) for the whole observation period ranges from 4.6-12.8% across all included counties. For the different phases, ranges are 4.1-16.7% (phase 2), 4.9-9.1% (phase 3a), 5.3-19.4% (phase 3b) and 5.4-19% (phase 4).

The ordinary least squares (OLS) model for the official active cases per 100000 students and teachers as reported by the KMK showed that in counties with a mask mandate during class in all types of schools (i.e. primary schools as well as secondary schools) the average number of weekly cases was lower by 56 per 100000 cases for both the students and teachers (Table 3). The mandate was associated with a greater reduction of infection activity among students and teachers than the total population. The mask mandate in class for all types of schools was associated with a reduction of case numbers relative to the general population of 29.8% for students and 24.3% for teachers (S11 Text, Table 1). Mandatory testing in schools in our study period was associated with an average increase of 50 notifications per 100000 per 14 days among students. (Table 2, S11 Text).
Urban counties were associated with a higher number of notified infections in students and a lower number in teachers compared to rural counties (S11 Text). A high degree of social deprivation in a

			county was associated with higher numbers of infections in teacher (S11 Text).<br>Not accounting for underestimation of infections, we found a high variability in the contribution of contacts from school infections to the overall transmission during the third wave from 2-12%. Accounting for age-specific underestimation of notified infections in comparison to actual infections based on seroprevalence estimates, this range was 2-13% (Fig.6).
	(*b*) Report category boundaries when continuous variables were categorized	S11 Text	S11 Text, Table 4 and 5 show previous analyses using the variable coding in Table 6. The original (full) model is presented next to the final model. The original selection of variables yielded some insensible and insignificant estimates due to multicollinearity (Table 7), thus some variables were excluded or rearranged for further analysis in the final model and the new variable grouping as per S3 Text, Table 1.
	(*c*) If relevant, consider translating estimates of relative risk into absolute risk for a meaningful time period		See point 16

Continued on next page

Other analyses	17	Report other analyses done—eg analyses of subgroups and interactions, and sensitivity analyses	S8 Text<br>S9 Text<br>S11 Text	e.g. S11 Text:<br>Sensitivity Analysis<br>S11 Text, Table 2 and 3 show the results of a sensitivity analysis splitting the observation period into rising infections during the 4th wave (W08-16) and waning infections during the 4th wave (W17-23).<br><br>S11 Text, Table 4 and 5 show previous analyses using the variable coding in Table 6. The original (full) model is presented next to the final model. The original selection of variables yielded some insensible and insignificant estimates due to multicollinearity (Table 7), thus some variables were excluded or rearranged for further analysis in the final model and the new variable grouping as per S3 Text, Table 1.
**Discussion**				
Key results	18	Summarise key results with reference to study objectives	Discussion	In this study of infection and transmission risks during the pandemic in Germany, we could show that the contribution of infection dynamics in schools to that of the general population was up to 13% under hygiene and testing measures. Furthermore, NPIs such as strict mask mandates (in all schools during class) and reduced presence models are associated with an effective reduction in infection and transmission risks in schools and their contribution to population infections.<br><br>We estimate a reduction by 24-30% of infection risk for both students and teachers relative to the population and by more than 55/100.000 per 14 days in absolute case numbers in those counties implementing mandatory surgical mask wearing during class in all schools.<br><br>These findings are reflected in our analysis on the contribution of schools to the general population infection dynamics. School vacation and closures reduced this contribution to as low as 2%. The peak at 12% contribution in W24 occurs with open schools, loosening population NPIs and loosening but still strict hygiene and testing measures in schools.

				Meanwhile, the lower peak at 7% early in phase 4 occurred during reduced attendance class models and stricter NPIs.<br><br>The majority of secondary cases of both students and staff happened in households. In schools, age-specific SARs show that transmission took place mostly from staff to staff and from student to student more than student to staff.<br><br>Most infections and transmission in school took place in phase 3a when schools operated in normal class models with some cloth mask rules and general hygiene concepts. Teachers showed a higher risk of infection than the general population. Infection risk for students increased with age.
Limitations	19	Discuss limitations of the study, taking into account sources of potential bias or imprecision. Discuss both direction and magnitude of any potential bias	Discussion<br>S12 Text	Limitations of our work are inherent in the notification process to both public health and educational agencies as well as the gathering of aggregate county-specific data (S12 Text). Both notification data itself as well as contact data is an underestimate of the actual infection dynamic. We attempted to account for underdetection with age-specific estimates taken from seroprevalence studies. This could make our estimate of contribution of school-contacts to overall transmission a potential underestimate. Residual confounding in the regression analysis is a possibility as we use aggregate county or school measures and did not have full access to individual-level confounding factors, e.g., on distribution of parental professions or industrial make-up of the counties included. We tried to include the most important confounding factors on infection dynamics in the population as well as deprivation and urbanity of counties in the analysis. SARs are limited by several factors. Contact tracing is inherently limited by both interviewer capacity and ability and interviewee memory and honesty, however with schools and households as contained domains the error margin is limited. Among contacts, the data did not indicate whether these were still susceptible to infection. However, it can be assumed that biases are similar in each region as they

are situated within the same country and timeline of events. A reduction in the proportion of the susceptible population through vaccination or infection is assumed relevant from March 2021, where it is included as a parameter in regression analysis. All considered, the SARs presented here described further transmission after cases have been detected in the school with limitations.

Limitations to the data are inherent in the process of disease reporting and contact tracing. Underdetection of cases, especially in the younger age group, will affect infection risk. Regional differences in infection environment and NPI adherence affect infection risk and make inter-regional comparison difficult. NPIs affecting domains of life other than schools were not considered and differed by federal state in Germany. R1, R2 and R4 collated data on students separately, with a margin of error in picking up the relevant classification of cases from contact tracing. R3 and R5 identified students based on age, thus older (in particular vocational) students will not be perfectly reflected in the analysis. Furthermore, health agencies collate cases based on registered place of residence. In particular R4 data will be affected by cross-border (country and county) commuters and administrative differentiation of cases by residence, as would be R1, R2 and R5 through peri-urban commuting.

Contact tracing fidelity depends on capacity and capability of health agencies, reporting by index cases and is inherently flawed. This is obvious in that a) the reproduction numbers for regions where full population datasets were provided (R3, R5) is below 1 and thus not compatible with the pandemic progression witnessed and b) on average, only 65% of index cases had any contacts reported at all (R1 54%, R3 54%, R4 42% and R5 84%), leading to a skewed picture of contacts and secondary attack rates as well as biases as to which index cases would have reported contacts and how or whether they would have been identified as secondary cases by authorities in due course. Furthermore,

especially for household cases the direction of transmission in instances of multiple household cases cannot always be clearly ascertained, the same holds true for attribution of origin of infected to any one contact, particularly during phases of high transmission. Among contacts, it was not possible to ascertain from the data whether these were still susceptible to infection. However, it can be assumed that biases are similar in each region as they are situated within the same state and timeline of events, and that a reduction in the proportion of the susceptible population through vaccination or infection is assumed relevant from March 2021, where it is included as a parameter in regression analysis. All considered, the SAR parameter describes further transmission after cases have been detected in the school with limitations.

Compared to health agencies, education agencies are not trained in contact tracing and systematic disease reporting, thus a greater margin of error is to be expected in those datasets gathered by education authorities.

A thorough interpretation of our results has to take into account significant limitations associated with our data. The regressions based on the KMK data span only a relatively narrow time window, which spans from calendar week 8-25 of 2021. Therefore, there may be time effects, which should be considered but cannot be included sufficiently in a statistical analysis. We tried to control for this to some extent by including the overall infection level in the local populace. Moreover, our intervention variables are obviously not ideal but rather a collection of either binary variables or approximations to include the differences in stringency of measures and attendance in school and roughly quantify the impacts these measures have on the infection risks of students and teachers as best as possible with limited data. As the NPIs have been introduced as bundles in most cases, we do not have a perfect experimental environment that would be needed for an optimal

				assessment of the NPIs' impacts on the infection risks. Therefore, the coefficients could be slightly biased in some cases as the Gauß-Markov assumptions, especially independence among the explanatory variables, are not completely fulfilled. We chose the variables in a manner that multicollinearity is minimised as well as possible, yet perfect independence is not given in the data. Another major limitation is the lack of comparability between the KMK and the RKI data. Whereas the RKI reports new cases weekly which can be stratified by age groups, the KMK reports known and current sick cases due to COVID-19. We tried to circumvent this limitation by comparing new cases of the previous two calendar weeks, as reported by the RKI, to the sick numbers reported by the KMK. The assumption behind this is that students or teachers currently excused from school due to an infection have been infected in one of the previous two weeks, which appears a reasonable assumption given the incubation time (13). Furthermore, KMK data do not discriminate by type of school on the district level and demographic data on the students and teachers is not reported. Finally, the KMK data may be vulnerable towards higher underdetection of COVID-19 cases, as students and teachers are not obligated to report their reason of absence correctly, i.e. it is not ensured that someone being excused from school will indeed report the real reason for their absence.
Interpretation	20	Give a cautious overall interpretation of results considering objectives, limitations, multiplicity of analyses, results from similar studies, and other relevant evidence	Discussion	Despite these limitations we conclude that school setting contribution to overall transmission in the population is relevant, but variable over different time periods in the pandemic and responsive to NPIs. In Germany, school-related NPIs, in particular masking and reduced attendance, have been successful in mitigating the spread of the virus among both students and teachers.
Generalisability	21	Discuss the generalisability (external validity) of the study results	Discussion<br>S12 Text	A thorough interpretation of our results has to take into account significant limitations associated with our data. The regressions based on the KMK data span only a relatively narrow time window, which spans from calendar week 8-25 of 2021. Therefore, there may be time effects, which should be considered but cannot be included sufficiently in a

			statistical analysis. We tried to control for this to some extent by including the overall infection level in the local populace. Moreover, our intervention variables are obviously not ideal but rather a collection of either binary variables or approximations to include the differences in stringency of measures and attendance in school and roughly quantify the impacts these measures have on the infection risks of students and teachers as best as possible with limited data. As the NPIs have been introduced as bundles in most cases, we do not have a perfect experimental environment that would be needed for an optimal assessment of the NPIs' impacts on the infection risks.<br><br>Contact tracing fidelity depends on capacity and capability of health agencies, reporting by index cases and is inherently flawed. This is obvious in that a) the reproduction numbers for regions where full population datasets were provided (R3, R5) is below 1 and thus not compatible with the pandemic progression witnessed and b) on average, only 65% of index cases had any contacts reported at all (R1 54%, R3 54%, R4 42% and R5 84%), leading to a skewed picture of contacts and secondary attack rates as well as biases as to which index cases would have reported contacts and how or whether they would have been identified as secondary cases by authorities in due course. Furthermore, especially for household cases the direction of transmission in instances of multiple household cases cannot always be clearly ascertained, the same holds true for attribution of origin of infected to any one contact, particularly during phases of high transmission.
**Other information**			
Funding	22	Give the source of funding and the role of the funders for the present study and, if applicable, for the original study on which the present article is based	This project received financial support by the Standing Conference of Ministers of Education and Cultural Affairs (KMK) in Germany. TH, BL, PV, IR, and GK received funding from the European Union's Horizon 2020 research and innovation program (101003480; Project CORESMA) and from the Initiative and Networking Fund of the

			Helmholtz Association. BL and SG received funding from the BMBF Netzwerk für Universitätsmedizin (NaFOUniMedCovid19, FKZ: 01KX202; Project COVIM). AJ, JD received funding from the BMBF (Bundesweites Forschungsnetz „Angewandte Surveillance und Testung“ (B-FAST); AP5: Anwendungsbereich Schulen und Kitas).

*Give information separately for cases and controls in case-control studies and, if applicable, for exposed and unexposed groups in cohort and cross-sectional studies.

Note: An Explanation and Elaboration article discusses each checklist item and gives methodological background and published examples of transparent reporting. The STROBE checklist is best used in conjunction with this article (freely available on the Web sites of PLoS Medicine at http://www.plosmedicine.org/, Annals of Internal Medicine at http://www.annals.org/, and Epidemiology at http://www.epidem.com/). Information on the STROBE Initiative is available at www.strobe-statement.org.
