# Supplementary material for: Infection and transmission risks of COVID-19 in schools and their contribution to population infections in Germany: A retrospective observational study using nationwide and regional health and education agency notification data
Source: PLoS Med. 2022 Dec 20;19(12):e1003913. doi: 10.1371/journal.pmed.1003913 (PMC9767368; doi:10.1371/journal.pmed.1003913)
Supplement: S1 Study Protocol — (DOCX) [file pmed.1003913.s003.docx]

# **Retrospective Observational Study on the Risk of SARS-CoV-2 Infections among Students and Staff**

# **in Schools and the Impact of Infection Control Measures**

# **Study protocol**

Study director: **Dr. med. Berit Lange**

**Helmholtz Centre for Infection Research (HZI)**

Inhoffenstr. 7,

D-38124 Braunschweig

Email: Berit.Lange@helmholtz-hzi.de

**Prof. Dr. Gerard Krause
Helmholtz Centre for Infection Research (HZI)**

Inhoffenstr. 7,

D-38124 Braunschweig

Tel.: +49 (0)531/6181-3101

E-Mail: [Gerard.Krause@helmholtz-hzi.de](mailto:Gerard.Krause@helmholtz-hzi.de)

| Sudip Jung Karki  **Scientist** | **Helmholtz Centre for Infection Research (HZI)**  Inhoffenstr. 7,  38124 Braunschweig  SudipJung.Karki@helmholtz-hzi.de |
| --- | --- |
| Torben Heinsohn  **Scientist** | Torben.Heinsohn@gstt.nhs.uk |

**Biometric analysis**

| Dr Barbora Kessel | **Helmholtz Centre for Infection Research GmbH**  Inhoffenstraße 7  38124 Braunschweig  E-Mail: Barbora.Kessel@helmholtz-hzi.de |
| --- | --- |

**Regulatory/corporate data protection representative**

| Harald Ohrdorf | **Helmholtz Centre for Infection Research GmbH**  Inhoffenstraße 7  38124 Braunschweig  Tel.: +49 531/6181-2050  E-Mail: [Datenschutzbeautragter@helmholtz-hzi.de](mailto:Datenschutzbeautragter@helmholtz-hzi.de) |
| --- | --- |

**Responsible for data processing**

| Silke Tannapfel  **Administrative Manager** | **Helmholtz Centre for Infection Research GmbH**  Inhoffenstraße 7  38124 Braunschweig  Tel.: +49 531/6181-2000,  E-Mail: [Silke.Tannapfel@helmholtz-hzi.de](mailto:Silke.Tannapfel@helmholtz-hzi.de) |
| --- | --- |

**Supporting institutions, companies, organisations**

For the data collection, cooperation is being entered into with various state authorities. Authorities interested are:

| School and Vocational Training Authority (Schulbehörde) Hamburg | Hamburger Straße 31  22083 Hamburg  Tel.: +49 40 428630 |
| --- | --- |
| Public Health Department Cologne | Neumarkt 15-21  50667 Köln  Tel.: +49 221 22133500 |
| Public Health Department Lübeck | Sophienstraße 2-8  23560 Lübeck  Tel.: +49 4511225315 |

**Funding**

| Helmholtz Centre for Infection Research (HZI) | The study is financed by third-party funding from the Helmholtz Association and the Standing Conference of the Ministers of Education and Cultural Affairs of the states. No funding was used for research by an institution in the commercial or profit-oriented sector. |
| --- | --- |

Table of Contents

[1 Background 4](#_Toc118296681)

[2 Aims of the Study 5](#_Toc118296682)

[3 Study Design and Methods 6](#_Toc118296683)

[3.1 Study design 6](#_Toc118296684)

[3.2 Data sources 6](#_Toc118296685)

[3.3 Population study 6](#_Toc118296686)

[3.3.1 Inclusion criteria 6](#_Toc118296687)

[3.3.2 Risks and benefits for the subjects 6](#_Toc118296688)

[3.3.3 Endpoints 6](#_Toc118296689)

[4 Data Collection and Analysis 8](#_Toc118296690)

[4.1 Data collection 8](#_Toc118296691)

[4.1.1 Data items 9](#_Toc118296692)

[4.2 Data analysis plan 14](#_Toc118296693)

[Objective 1 15](#_Toc118296694)

[Objective 2 15](#_Toc118296695)

[Objective 3 15](#_Toc118296696)

[5 Data Protection 16](#_Toc118296697)

[6 References 16](#_Toc118296698)

# 1 Background

Coronavirus-Disease-19, commonly known as COVID-19, is a global public health threat caused by severe acute respiratory syndrome coronavirus-2 (SARA-CoV-2) (1). Since its outbreak in late December 2019 in Wuhan, China (2), it has affected more than 200 countries with 86 million (M) cases and was responsible for 1.8 M deaths worldwide (3, 4). This infection affects people of all ages. The severity increases with age (more severe in people >60 years old), with the elderly having more symptomatic cases compared to adults and children. Adults and children show few symptoms. Similarly, children show a mild clinical course and a less severe outcome compared to adults and the elderly (5-8).

In Germany, the first cases of SARS-CoV-2 were reported in January 2020, one month after the start of spread in Germany (9). The available data on the spread of COVID-19 are based on the COVID-19 cases (also called case report data) reported by the health authorities according to the Infection Protection Act (IfSG). Cases are detected by direct virus detection via nasopharyngeal swabs using PCR or by cultural isolation of the pathogen (10). However, an unknown proportion of the infected persons are asymptomatic (11-13) and many subclinical or mild infections are not included in the reporting statistics as confirmed cases.

Most countries rely on non-pharmaceutical interventions such as isolation, quarantine, and social distancing to contain COVID-19 transmission. With limited specific therapeutic interventions and limited vaccination, the public health response includes enforced lockdowns and large-scale school closures, which are chosen worldwide to curb the transmission of the pathogen (6, 14, 15). Also, school closures also began in early March in Germany (16).

Studies regarding the susceptibility of children to SARS-CoV-2 infection suggest that children are less to equally susceptible than adults (17-21) (22, 23). They are somewhat equally infectious and usually have more contacts than adults. For example, studies from contact tracing data from Israel, India, Italy and South Korea suggest that children over 10 years of age can be as infectious as adults (18, 23-25) and studies measuring viral load excretion in children and adults show them to be similar to adults (26, 27). The extent to which symptomatic or asymptomatic paediatric COVID-19 cases contribute to community transmission is largely unclear (28, 29). The prospective studies with data limited to prolonged school closures suggest that infection in children is mainly asymptomatic and transmitted through household members (30-32). Thus, educational institutions are a relevant and, probably regionally, a very heterogeneous part of the infection occurrence.

Since long-term school closure leads to disruption of children’s education, has a negative impact on children’s overall development, affects mental health and nutrition, and increases ethic and socio-economic disparities in school performance (33-38), on the other hand, many efforts have been made to keep schools open as long as possible – even with increased infection rates – through high infection control measures. So far, it is unclear how well these measures have worked regionally in each case, and in Germany there is no study to date that correlates data from different regions with different such regulations with the contribution of schools to the incidence of infections. We would therefore like to retrospectively investigate – also in order to learn for future epidemics and pandemics – the risk of infection of children and school staff, transmission within and through school as well as the effect of infection protection measures in school in different regions in Germany.

# 2 Aims of the Study

The overall aim of this retrospective observational study is to compare the risk of infection by SARS-CoV-2 in children and school staff with the general population over the year. Similarly, this study aims to find out what proportion of transmission in the general population affects students and school staff. And ultimately, we would also like to know whether infection control measures have an impact on these parameters. The following specific objectives are pursued in this study:

1. Description of the risk of infection by SARS-CoV-2 for pupils and staff compared to the general population over the study interval
2. Description of transmission in and through schools over the study interval
3. Description and effectiveness of infection control measures in schools on objectives 1 and 2

# 3 Study Design and Methods

## 3.1 Study design

This is a retrospective observational study. In this study, we retrospectively evaluated prospectively collected SARS-CoV-2 infection reporting data from selected health and school authorities in Germany. The case report data collected from February 2020 to February 2021 is used to answer the study questions. Different geographical, infection epidemiological and regulatory regions are selected among the selected school and health authorities.

## 3.2 Data sources

We analysed laboratory-confirmed COVID-19 case reporting data from the national surveillance system from February 2020 to February 2021. COVID-19 cases are confirmed either by detection of SARS-CoV-2 nucleic acid by PCR in a respiratory specimen or by cultural isolation of the pathogen from the specimen. The confirmed cases are reported by the doctors and laboratories to the local health authorities. The data are then forwarded by the respective state health authorities to the Robert Koch Institute (16).

The case registration data are linked to the nationwide resident data from the Residents' Registration Office in Germany. The Residents' Registration Office in Germany registers every resident domiciled in the country.

For the data collection in this study, data from selected health offices in Germany are provided. These data are anonymised - personal data are not collected.

## 3.3 Population study

### 3.3.1 Inclusion criteria

Individuals with confirmed SARS-CoV-2 infection and their contacts reported to public health departments from February 2020 to February 2021 are included in this study. The data are collected anonymously.

### 3.3.2 Risks and benefits for the subjects

This study is a retrospective observational study based on anonymised data with no risks or direct benefits to the subjects. The benefit of this study is to gain knowledge about the risk of infection in schools, its regional variability and its value as part of the evidence base for recommendations on infection control measures, especially for schools.

### 3.3.3 Endpoints

The main objective of this retrospective observational study is to compare the risk of infection from COVID-19 cases among school staff and children with the general population (objective 1) and to measure the extent to which school staff and pupils transmit the infection to contacts in the home and school (objective 2). It also investigates how effective infection control measures in schools are in reducing the risk of infection and reducing transmission within schools and households (objective 3).

The following **endpoints** are collected according to the above study objectives:

1. Hazard ratio with 95% confidence interval of infection rate of pupils and school staff/risk of infection of comparable infection rate of population control groups over the study interval
2. Secondary attack rates in schools and in the home with 95% confidence interval over the study interval
3. Influence of implementation and termination of infection control measures in schools on hazard ratio and secondary attack rates

# 4 Data Collection and Analysis

## 4.1 Data collection

We analysed laboratory-confirmed COVID-19 case reporting data from the national surveillance system from February 2020 to February 2021. COVID-19 cases are confirmed either by detection of SARS-CoV-2 nucleic acid by PCR in a respiratory sample or by cultural isolation of the pathogen from the sample. Doctors and laboratories report confirmed cases to local health authorities. The respective state health authorities then forward the data to the Robert Koch Institute.

For data collection, we worked with selected health authorities and school authorities. For this purpose, in consultation with interested health authorities and school authorities, we have created minimal data sets and extracted them from existing data in close cooperation with the health authorities. The formed dataset contains data on four main categories, namely reported cases, contacts, number of tests performed and context of intervention.

We extracted the following data in the four categories:

1. Data on reported cases
   - Age, gender and comorbidities
   - Known infection context of the case, part of an outbreak
   - Symptoms, hospitalisations, deaths
   - Date of diagnosis, dates of hospitalisation, date of death
   - Number of contacts in household (children <10 years, children aged 10-18 years, adults), school (pupils/staff), work and other contact areas.
   - Number of infected contacts in the household (children <10 years, children of age 10-18 years, adults), at school (pupils/staff), at work and in other contact areas
2. Contact data
   - Age, gender and comorbidities
   - Contact areas: Household, school, work and other contact areas
   - Date and results of the tests carried out
3. Number of tests performed for SARS-CoV-2
   - Number of PCR tests performed
   - Number of antigen tests performed
   - Testing of pupils and school staff
   - Tests in different age groups
4. Context of measures
   - Data on specific interventions
   - Regional protection measures for schools (implementation period and school type and application)

Our aim is to look at the relative and absolute risk difference stratified by age, gender and federal state in Germany over the last year.

**Table 1: Endpoints and Outcomes**

| Objectives | Endpoints | Results |
| --- | --- | --- |
| 1. Risk of COVID-19 for children and staff compared to the general population | - Risk or COVID-19 among children and school staff and in the general population, stratified by age group, sex and federal state | - Hazard ratio with 95% confidence interval of infection rate of pupils and school staff/risk of infection of comparable infection rate of population control groups over time |
| 1. Transmission in and through schools over the study interval | - Secondary attack rates in schools and households | - Secondary attack rates (proportion of positive contacts/contacts) in schools and in the home with 95% confidence interval over time |
| 1. Description and effectiveness of interventions that reduce 1 & 2 | - Influence of the implementation and termination of infection control measures in schools on the risk of infection and transmission of pupils and school staff | - Adjusted hazard ratio with 95% confidence interval of infection rate of pupils and school staff/risk of infection of comparable infection rate of population control groups over the time before and after infection control measures - Secondary attack rates before and after infection control measures |

### 4.1.1 Data items

The following data are collected in detail – if available – in cooperation with health offices and school authorities for the study interval.

**Table 2: Data items and sources**

| **Category** | **Data item** | **Data source** | **Description** |
| --- | --- | --- | --- |
| Case | Datum_Diagnose [Date_Diagnosis] | Public health department/ school authority | Date of the positive test result |
|  | Datum_Meldung [Date_Notifification] | Public health department/ school authority | Date of notification of the infection at the public health department |
|  | Alter [Age] | Public health department/ school authority | In years |
|  | Geschlecht  [Sex] | Public health department/ school authority | In m / w / d |
|  | Komorbidität _bin  [Comorbidity_binary] | Public health department/ school authority | Yes / no |
|  | Komorbidität _kat  [Comorbidity_category] | Public health department/ school authority | According to system and risk |
|  | Komorbidität _det  [Comorbiditry_detail] | Public health department/ school authority | Detailed performance |
|  | Infektionsumfeld_kat  [Infectionenvironment_category] | Public health department/ school authority | e.g., school, household, leisure |
|  | Infektionsumfeld_det  [Infectionenvironment_detail] | Public health department/ school authority | Description |
|  | Teil eines Ausbruches_bin  [Partofooutbreak_binary | Public health department/ school authority | Yes / no |
|  | Symptome_bin  [Symptoms_binary] | Public health department/ school authority | Yes / no |
|  | Symptome_kat  [Symptoms_category] | Public health department/ school authority | Specific, non-specific |
|  | Symptome_det  [Symptoms_detail] | Public health department/ school authority | Description, intensity, duration |
|  | Hospitalisierung_bin  [Hospitalisation_binary] | Public health department/ school authority | Yes / no |
|  | Datum Hospitalisierung  [Date Hospitalisation] | Public health department/ school authority | Date of recording |
|  | Tod_bin  [Death_binary] | Public health department/ school authority | Yes / no |
|  | Datum Tod  [Date Death] | Public health department/ school authority | Date of death |
|  | Genesung_bin  [Recovery_binary] | Public health department/ school authority | Yes / no |
|  | Datum Genesung  [Date Recovery] | Public health department/ school authority | Date of discharge from hospital |
|  | Anzahl Kontakte_ges  [Number Contacts_total] | Public health department/ school authority | Number of reported contacts |
|  | Anzahl Kontakte_Schule  [Number Contacts_School] | Public health department/ school authority | Number of reported contacts from the school environment |
|  | Anzahl Kontakte_Schule_Kindbis10  [Number Contacts_School_Childto10] | Public health department/ school authority | Number of reported contacts under 10 years in the school environment |
|  | Anzahl Kontakte_Schule_Kindbis18  [Number Contacts_School_Childto18] | Public health department/ school authority | Number of reported contacts from 10-18 years in the school environment |
|  | Anzahl Kontakte_Schule_Erwachsene  [Number Contacts_School_Adults] | Public health department/ school authority | Number of reported contacts over 18 in the school environment |
|  | Anzahl_Kontakte_Haushalt  [Number_Contacts_Household] | Public health department/ school authority | Number of reported contacts in the household |
|  | Anzahl Kontakte_Haushalt_Kindbis10 [Number Contacts_Household_Childto10] | Public health department/ school authority | Number of reported contacts under 10 years in the household |
|  | Anzahl Kontakte_Haushalt_Kindbis18  [Number Contacts_Household_Childto18] | Public health department/ school authority | Number of reported contacts aged 10-18 in the household |
|  | Anzahl Kontakte_Haushalt_Erwachsene  [Number Contacts_Household_Adults] | Public health department/ school authority | Number of reported contacts over 18 in the household |
|  | Anzahl_Kontakte_anderes  [Number_Contacts_other] | Public health department/ school authority | Number of reported contacts in leisure time, etc., based on categories of the Robert Koch Institute |
|  | Anzahl_Kontakt_pos_ges  [Number_Contacts_positive_total] | Public health department/ school authority | Number of contacts tested positive |
|  | Anzahl_Kontakte_pos_Schule [Number_Contacts_positive_School] | Public health department/ school authority | Number of positively tested contacts from the school environment |
|  | Anzahl Kontakte_Schule_pos_Kindbis10  [Number Contacts_School_positive_Childto10] | Public health department/ school authority | Number of positively tested contacts under 10 years in the school environment |
|  | Anzahl Kontakte_Schule_pos_Kindbis18  [Number Contacts_School_positive_Childto18] | Public health department/ school authority | Number of positively tested contacts from 10-18 years in the school environment |
|  | Anzahl Kontakte_Schule_pos_Erwachsene  [Number Contacts_School_positive_Adults] | Public health department/ school authority | Number of positively tested contacts over the age of 18 in the school environment |
|  | Anzahl_Kontakte_pos_Haushalt  [Number Contacts_positive_Household] | Public health department/ school authority | Number of positively tested contacts in the household |
|  | Anzahl Kontakte_Haushalt_pos_Kindbis10  [Number Contacts_Household_positive_Childto10] | Public health department/ school authority | Number of positively tested contacts under 10 years in the household |
|  | Anzahl Kontakte_Haushalt_pos_Kindbis18  [Number Contacts_Household_Childto18] | Public health department/ school authority | Number of positively tested contacts from 10-18 years in the household |
|  | Anzahl Kontakte_Haushalt_pos_Erwachsene  [Number Contacts_Household_positive_Adults] | Public health department/ school authority | Number of positively tested contacts over 18 years in the household |
|  | Anzahl_Kontakte_pos_anderes  [Number_Contacts_positive_other] | Public health department/ school authority | Number of positively tested contacts in leisure time, etc., based on categories of the Robert Koch Institute |
| Contacts | Alter [Age] | Public health department/ school authority | In years |
|  | Geschlecht  [Sex] | Public health department/ school authority | m / w / d |
|  | Komorbidität _bin  [Comorbidity_binary] | Public health department/ school authority | Yes / no |
|  | Komorbidität _kat  [Comorbidity_category] | Public health department/ school authority | According to system and risk |
|  | Komorbidität _det  [Comorbiditry_detail] | Public health department/ school authority | Detailed performance |
|  | Infektionsumfeld_kat  [Infectionenvironment_category] | Public health department/ school authority | e.g. school, household, leisure |
|  | Infektionsumfeld_det  [Infectionenvironment_detail] | Public health department/ school authority | Description of the infection environment |
| Number of tests | Anzahl_PCR_ges/Woche  [Number_PCR_total/Week] | Laboratory/ Public health department | Number of PCR tests performed per week |
|  | Anzahl_Antigen_ges/Woche  [Number_Antigen_total/Week] | Laboratory/ Public health department | Number of antigen tests performed per week |
|  | Anzahl PCR_Altersgruppen/Woche  [Number PCR_Agegroups/Week] | Laboratory/ Public health department | Number of PCR tests performed per week by age group |
|  | Anzahl_Antigen_Altersgruppen/Woche  [Number_Antigen_Agegroups/Week] | Laboratory/ Public health department | Number of antigen tests performed per week by age group |
|  | Anzahl_PCR_Schulpersonal/Woche  [Number_PCR_Schoolstaff/Week] | Laboratory/ Public health department | Number of PCR tests performed per week on school staff |
|  | Anzahl_Antigen_Schulpersonal/Woche  [Number_Antigen_Schoolstaff/Week] | Laboratory/ Public health department | Number of antigen tests carried out per week on school staff |
|  | Anzahl_PCR_SchülerInnen/Woche  [Number_PCR_Students/Week] | Laboratory/ Public health department | Number of PCR tests carried out on pupils per week |
|  | Anzahl_Antigen_SchülerInnen/Woche  [Number_Antigen_Students/Week] | Laboratory/ Public health department | Number of antigen tests carried out on pupils per week |
| Interventions | Infektionsschutzmaßnahmen_infektionsdynamisch_angepasst  [infectionmeasures_infectiondynamically_adjusted] | Public health department | Description of the ISM and its application |
|  | ISM_MNS_Grundschulen Datum Implementierung  [InfectionControlMeasure(ICM)_Masks_Primary school Date Implementation] | Public health department | Date of implementation of ISM and its application in primary schools |
|  | ISM_MNS_Grundschulen Datum Beenden  [ICM_Masks_Primary school Date Termination] | Public health department | Date of repeal of ISM and its application in primary schools |
|  | ISM_MNS_Grundschule Det  [ICM_Masks_Primary school Details] | Public health department | Description of ISM and its application in primary schools |
|  | ISM_MNS_weiterführend Datum Implementierung  [ICM_Masks_Secondary school Date Implementation] | Public health department | Date of implementation of ISM and its application in secondary schools |
|  | ISM_MNS_weiterführend Datum Beenden  [ICM_Masks_Secondary school Date Termination] | Public health department | Date of repeal of ISM and its application in secondary schools |
|  | ISM_MNS_weiterführend Det  [ICM_Masks_Secondary school Details] | Public health department | Description of ISM and its application in secondary schools |
|  | ISM_Kohortierung_Grundschule Datum Implementierung  [ICM_Cohorting_Primary school Date Implementation] | Public health department | Date of implementation of cohorting in primary schools |
|  | ISM_Kohortierung_Grundschule Datum Beenden  [ICM_Cohorting_Primary school Date Termination] | Public health department | Date of abolition of cohorting in primary schools |
|  | ISM_Kohortierung_Grundschule Det  [ICM_Cohorting_Primary school Details] | Public health department | Description of cohorting in primary schools |
|  | ISM_Kohortierung_weiterführend Datum Implementierung  [ICM_Cohorting_Secondary school Date Implementation] | Public health department | Date of implementation of cohorting in secondary schools |
|  | ISM_Kohortierung_weiterführend Datum Beenden  [ICM_Cohorting_Secondary school Date Termination] | Public health department | Date of abolition of cohorting in secondary schools |
|  | ISM_Kohortierung_weiterführend Det  [ICM_Cohorting_Secondary school Details] | Public health department | Description of cohorting in secondary schools |
|  | ISM_MaßnahmenSchulweg_Grundschule Datum Implementierung  [ICM_MeasuresCommute_Primary school Date Implementation] | Public health department | Date of implementation of measures concerning the way to school in primary schools |
|  | ISM_MaßnahmenSchulweg_Grundschule Datum Beenden  [ICM_MeasuresCommute_Primary school Date Termination] | Public health department | Date of lifting of measures concerning the way to school in primary schools |
|  | ISM_MaßnahmenSchulweg_Grundschule Det  [ICM_MeasuresCommute_Primary school Details] | Public health department | Description of measures concerning the way to school in primary schools |
|  | ISM_MaßnahmenSchulweg_weiterführend Datum Implementierung  [ICM_MeasuresCommute_Secondary school Date Implementation] | Public health department | Date of implementation of measures concerning the way to school in secondary schools |
|  | ISM_MaßnahmenSchulweg_weiterführend Datum Beenden  [ICM_MeasuresCommute_Secondary school Date Termination] | Public health department | Date of lifting of measures concerning the way to school in secondary schools |
|  | ISM_MaßnahmenSchulweg_weiterführend Det  [ICM_MeasuresCommute_Secondary school Details] | Public health department | Description of the measures  concerning the way to school in secondary schools |
|  | ISM_Screening_Grundschule Datum Implementierung  [ICM_Screening_Primary school Date Implementation] | Public health department | Date of implementation of screening in primary schools |
|  | ISM_Screening_Grundschule Datum Beenden  [ICM_Screening_Primary school Date Termination] | Public health department | Date of completion of screening in primary schools |
|  | ISM_Screening_Grundschule Det  [ICM_Screening_Primary school Details] | Public health department | Description of screening in primary schools |
|  | ISM_Screening_weiterführend Datum Implementierung  [ICM_Screening_Secondary school Date Implementation] | Public health department | Date of implementation of screening in secondary schools |
|  | ISM_Screening_weiterführend Datum Beenden  [ICM_Screening_Secondary school Date Termination] | Public health department | Date of completion of screening in secondary schools |
|  | ISM_Screening_weiterführend Det  [ICM_Screening_Secondary school Details | Public health department | Description of the screening in secondary schools |

## 4.2 Data analysis plan

The statistical analysis is carried out with STATA and R.

Demographic characteristics are summarised. Continuous variables are given as median and interquartile range (IQR). Categorical variable is given as number and percentage.

### Objective 1

**Comparison of the risk of infection of COVID-19 cases in school staff and children with the general population over the study interval**

First, infection rates among pupils and school staff are described over time. Where possible, regional, age-matched control groups (school staff, older pupils) are formed; where this is not possible, infection rates in the population are described as a comparison. In adequate survival analyses, hazard ratios adjusted for test rates are calculated with 95% confidence intervals of the infection rate of pupils and school staff/risk of infection of comparable infection rate of population control groups.

### Objective 2

**Transmission in and through schools over time**

We described the change in regional secondary attack rates in schools and in the household by and from students and school staff over the year and examine it in intermittent time series analyses. Monthly data from February 2020 to February 2021 is used for this purpose.

### Objective 3

**Description and effectiveness of interventions that reduce infection risk and transmission**

First, we described implemented infection control measures per region with implementation and time period. We examined the absolute difference and the ratio and 95% confidence interval of hazard ratios of infection rates and secondary attack rates before and after the intervention with suitable regression models.

# 5 Data Protection

The necessary measures for data protection and information technology (IT) security were defined in a data protection concept for the study. This concept was coordinated with the data protection officer of the HZI. The data protection concept was drawn up in accordance with the requirements of the General Data Protection Regulation (GDPR) and the latest revision of the Federal Data Protection Act (25 May 2018). The study involves data processing for research in the public interest (§ 89 GDPR). The lawfulness of the processing results from GDPR Art. 6(1)(a). The lawfulness of the cooperation with the public health department on the basis of the Infection Protection Act results from GDPR Art.6(1)(c).

When determining the organisational, technical and personnel measures to be implemented for data protection and IT security, the relevant standards of the Federal Office for Information Security as well as the guidelines and recommendations for safeguarding Good Epidemiological Practice are taken into account.

A data use contract in accordance with the European General Data Protection Regulation (GDPR) is concluded with each health department and school authority.

Data protection and IT security risks are mitigated as follows: The data are anonymised before it is received at the HZI. They do not allow any conclusions to be drawn about individuals. The storage of medical data (test results, treatment data from doctors, hospitals, health facilities or health authorities) is always anonymised.

After assessing the individual risks, the threat analysis came to the conclusion that the subjects concerned have a low risk that their data could be misused due to the technical and organisational measures that have been put in place.

The research data are collected at the HZI and stored for 10 years. The data are stored in a protected database to which only members of the study team, who are all bound by professional/data secrecy, have access.

The data collected during the study can also be used and processed anonymously for future research projects.

# 6 References

1. Wang M, Liao Z. SARS-CoV-2 and COVID-19: How much do we know? Acta virologica. 2020;64(3):288-96.

2. Yousefi B, Valizadeh S, Ghaffari H, Vahedi A, Karbalaei M, Eslami M. A global treatments for coronaviruses including COVID-19. Journal of cellular physiology. 2020;235(12):9133-42.

3. Worldometer. COVID-19 CORONA VIRUS/DEATH TOLL: Worldometer; 2021 [updated 05.01.2021. Available from: <https://www.worldometers.info/coronavirus/coronavirus-death-toll/>.

4. Worldometer. COVID-19 CORONA VIRUS PANDEMIC: Woldometer; [cited 2021 05.01.2021]. Available from: <https://www.worldometers.info/coronavirus/>.

5. Wu Z, McGoogan JM. Characteristics of and Important Lessons From the Coronavirus Disease 2019 (COVID-19) Outbreak in China: Summary of a Report of 72 314 Cases From the Chinese Center for Disease Control and Prevention. JAMA. 2020;323(13):1239-42.

6. McCloskey B, Heymann DL. SARS to novel coronavirus – old lessons and new lessons.

7. Leung C. Clinical characteristics of COVID-19 in children: Are they similar to those of SARS? Pediatric pulmonology. 2020;55(7):1592-7.

8. Patel NA. Pediatric COVID-19: Systematic review of the literature. American journal of otolaryngology. 2020;41(5):102573.

9. Böhmer MM, Buchholz U, Corman VM, Hoch M, Katz K, Marosevic DV, et al. Investigation of a COVID-19 outbreak in Germany resulting from a single travel-associated primary case: a case series. The Lancet Infectious diseases. 2020;20(8):920-8.

10. RKI. COVID-19: Fallzahlen in Deutschland und weltweit. 2020.

11. Chan JF, Yuan S, Kok KH, To KK, Chu H, Yang J, et al. A familial cluster of pneumonia associated with the 2019 novel coronavirus indicating person-to-person transmission: a study of a family cluster. Lancet. 2020;395(10223):514-23.

12. Rothe C, Schunk M, Sothmann P, Bretzel G, Froeschl G, Wallrauch C, et al. Transmission of 2019-nCoV Infection from an Asymptomatic Contact in Germany. N Engl J Med. 2020;382(10):970-1.

13. Pan X, Chen D, Xia Y, Wu X, Li T, Ou X, et al. Asymptomatic cases in a family cluster with SARS-CoV-2 infection. Lancet Infect Dis. 2020;20(4):410-1.

14. Anderson RM, Heesterbeek H, Klinkenberg D, Hollingsworth TD. How will country-based mitigation measures influence the course of the COVID-19 epidemic? Lancet (London, England). 2020;395(10228):931-4.

15. Wilder-Smith A, Freedman DO. Isolation, quarantine, social distancing and community containment: pivotal role for old-style public health measures in the novel coronavirus (2019-nCoV) outbreak. Journal of Travel Medicine. 2020;27(2).

16. Otte Im Kampe E, Lehfeld AS, Buda S, Buchholz U, Haas W. Surveillance of COVID-19 school outbreaks, Germany, March to August 2020. Euro surveillance : bulletin Europeen sur les maladies transmissibles = European communicable disease bulletin. 2020;25(38).

17. Goldstein E, Lipsitch M, Cevik M. On the effect of age on the transmission of SARS-CoV-2 in households, schools and the community. The Journal of infectious diseases. 2020.

18. Dattner I, Goldberg Y, Katriel G, Yaari R, Gal N, Miron Y, et al. The role of children in the spread of COVID-19: Using household data from Bnei Brak, Israel, to estimate the relative susceptibility and infectivity of children. medRxiv. 2020:2020.06.03.20121145.

19. Davies NG, Klepac P, Liu Y, Prem K, Jit M, Eggo RM. Age-dependent effects in the transmission and control of COVID-19 epidemics. Nature medicine. 2020;26(8):1205-11.

20. Dong Y, Mo X, Hu Y, Qi X, Jiang F, Jiang Z, et al. Epidemiology of COVID-19 Among Children in China. Pediatrics. 2020;145(6).

21. Jing Q-L, Liu M-J, Zhang Z-B, Fang L-Q, Yuan J, Zhang A-R, et al. Household secondary attack rate of COVID-19 and associated determinants in Guangzhou, China: a retrospective cohort study. The Lancet Infectious Diseases. 2020;20(10):1141-50.

22. Bi Q, Wu Y, Mei S, Ye C, Zou X, Zhang Z, et al. Epidemiology and transmission of COVID-19 in 391 cases and 1286 of their close contacts in Shenzhen, China: a retrospective cohort study. The Lancet Infectious diseases. 2020;20(8):911-9.

23. Laxminarayan R, Wahl B, Dudala SR, Gopal K, Mohan C, Neelima S, et al. Epidemiology and transmission dynamics of COVID-19 in two Indian states. medRxiv. 2020:2020.07.14.20153643.

24. Fateh-Moghadam P, Battisti L, Molinaro S, Fontanari S, Dallago G, Binkin N, et al. Contact tracing during Phase I of the COVID-19 pandemic in the Province of Trento, Italy: key findings and recommendations. medRxiv. 2020:2020.07.16.20127357.

25. Park YJ, Choe YJ, Park O, Park SY, Kim YM, Kim J, et al. Contact Tracing during Coronavirus Disease Outbreak, South Korea, 2020. Emerging infectious diseases. 2020;26(10):2465-8.

26. Jones TC, Mühlemann B, Veith T, Biele G, Zuchowski M, Hofmann J, et al. An analysis of SARS-CoV-2 viral load by patient age. medRxiv. 2020:2020.06.08.20125484.

27. L'Huillier AG, Torriani G, Pigny F, Kaiser L, Eckerle I. Culture-Competent SARS-CoV-2 in Nasopharynx of Symptomatic Neonates, Children, and Adolescents. Emerging infectious diseases. 2020;26(10):2494-7.

28. Lavezzo E, Franchin E, Ciavarella C, Cuomo-Dannenburg G, Barzon L, Del Vecchio C, et al. Suppression of a SARS-CoV-2 outbreak in the Italian municipality of Vo'. Nature. 2020;584(7821):425-9.

29. Vermund SH, Pitzer VE. Asymptomatic transmission and the infection fatality risk for COVID-19: Implications for school reopening. Clinical infectious diseases : an official publication of the Infectious Diseases Society of America. 2020.

30. Posfay-Barbe KM, Wagner N, Gauthey M, Moussaoui D, Loevy N, Diana A, et al. COVID-19 in Children and the Dynamics of Infection in Families. Pediatrics. 2020;146(2).

31. Qiu H, Wu J, Hong L, Luo Y, Song Q, Chen D. Clinical and epidemiological features of 36 children with coronavirus disease 2019 (COVID-19) in Zhejiang, China: an observational cohort study. The Lancet Infectious diseases. 2020;20(6):689-96.

32. Zimmermann P, Curtis N. Coronavirus Infections in Children Including COVID-19: An Overview of the Epidemiology, Clinical Features, Diagnosis, Treatment and Prevention Options in Children. The Pediatric infectious disease journal. 2020;39(5):355-68.

33. Armitage R, Nellums LB. Considering inequalities in the school closure response to COVID-19. The Lancet Global health. 2020;8(5):e644.

34. Dunn CG, Kenney E, Fleischhacker SE, Bleich SN. Feeding Low-Income Children during the Covid-19 Pandemic. The New England journal of medicine. 2020;382(18):e40.

35. Levinson M, Cevik M, Lipsitch M. Reopening Primary Schools during the Pandemic. The New England journal of medicine. 2020;383(10):981-5.

36. Van Lancker W, Parolin Z. COVID-19, school closures, and child poverty: a social crisis in the making. The Lancet Public health. 2020;5(5):e243-e4.

37. Yoshikawa H, Wuermli AJ, Britto PR, Dreyer B, Leckman JF, Lye SJ, et al. Effects of the Global Coronavirus Disease-2019 Pandemic on Early Childhood Development: Short- and Long-Term Risks and Mitigating Program and Policy Actions. The Journal of pediatrics. 2020;223:188-93.

38. Viner RM, Russell SJ, Croker H, Packer J, Ward J, Stansfield C, et al. School closure and management practices during coronavirus outbreaks including COVID-19: a rapid systematic review. The Lancet Child & adolescent health. 2020;4(5):397-404.
